# Peculiarities of Phase Formation in Mn-Based Na SuperIonic Conductor (NaSICon) Systems: The Case of Na_1+2*x*_Mn_*x*_Ti_2–*x*_(PO_4_)_3_ (0.0 ≤ *x* ≤ 1.5)

**DOI:** 10.1021/acs.chemmater.1c02775

**Published:** 2021-10-21

**Authors:** Gustautas Snarskis, Jurgis Pilipavičius, Denis Gryaznov, Lina Mikoliu̅naitė, Linas Vilčiauskas

**Affiliations:** † Center for Physical Sciences and Technology (FTMC), Saulėtekio al. 3, LT-10257 Vilnius, Lithuania; ‡ Institute of Solid State Physics, University of Latvia, Kengaraga 8, LV-1063 Riga, Latvia; § Institute of Chemistry, Vilnius University, Saulėtekio al. 3, LT-10257 Vilnius, Lithuania

## Abstract

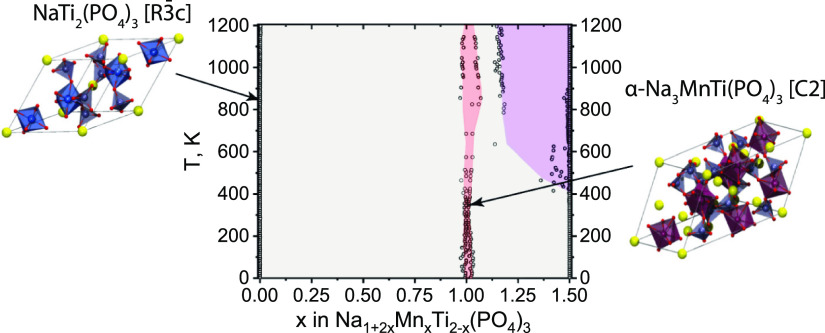

NAtrium SuperIonic CONductor (NASICON) structured phosphate framework compounds are attracting a great deal of interest as suitable electrode materials for “rocking chair” type batteries. Manganese-based electrode materials are among the most favored due to their superior stability, resource non-criticality, and high electrode potentials. Although a large share of research was devoted to Mn-based oxides for Li- and Na-ion batteries, the understanding of thermodynamics and phase formation in Mn-rich polyanions is still generally lacking. In this study, we investigate a bifunctional Na-ion battery electrode system based on NASICON-structured Na_1+2*x*_Mn_*x*_Ti_2–*x*_(PO_4_)_3_ (0.0 ≤ *x* ≤ 1.5). In order to analyze the thermodynamic and phase formation properties, we construct a composition–temperature phase diagram using a computational sampling by density functional theory, cluster expansion, and semi-grand canonical Monte Carlo methods. The results indicate finite thermodynamic limits of possible Mn concentrations in this system, which are primarily determined by the phase separation into stoichiometric Na_3_MnTi(PO_4_)_3_ (*x* = 1.0) and NaTi_2_(PO_4_)_3_ for *x* < 1.0 or NaMnPO_4_ for *x* > 1.0. The theoretical predictions are corroborated by experiments obtained using X-ray diffraction and Raman spectroscopy on solid-state and sol–gel prepared samples. The results confirm that this system does not show a solid solution type behavior but phase-separates into thermodynamically more stable sodium ordered monoclinic α-Na_3_MnTi(PO_4_)_3_ (space group *C*2) and other phases. In addition to sodium ordering, the anti-bonding character of the Mn–O bond as compared to Ti–O is suggested as another important factor governing the stability of Mn-based NASICONs. We believe that these results will not only clarify some important questions regarding the thermodynamic properties of NASICON frameworks but will also be helpful for a more general understanding of polyanionic systems.

## Introduction

The search and design of suitable electrode materials remains one of the major challenges for the future development of battery technologies. To a large extent, the properties of electrode active materials dictate the key characteristics of an entire battery system. For instance, in a typical Li-ion battery cell, the properties of electrode materials such as charge capacity and energy density are responsible for ∼50–70% of the cell energy density and cost, whereas materials stability strongly affects the cell lifetime.^1−6^ NAtrium SuperIonic CONductor (NASICON)-type framework compounds, originally sought as suitable solid electrolytes for high-temperature liquid sodium–sulfur batteries,^7^ are attracting increasing attention as potent materials for Na-ion insertion (“rocking chair”) battery (NIB) electrodes.^8,9^ Presently, NASICON-structured phosphates with a general formula of Na_1–4_M′M″(PO_4_)_3_ are probably the most studied and applied polyanion electrode materials for NIBs.^8,9^ They show a number of advantages over other materials by providing a very stable and robust framework with fast ion insertion kinetics and three-dimensional bulk mobility as well as wide selection of electrode redox potentials. The latter can be achieved by selecting an appropriate metal redox couple or their combination (i.e., M′, M″ = Ti, Mn, V, Cr, Fe, etc.).^10,11^ In addition to the possibility of tuning electrode potential, metal mixing and substitution can also increase stability, suppress Jahn–Teller distortions, or even provide an ability to serve as both a positive and a negative electrode material at the same time.^12−16^

Mn-based oxide and polyanion materials are among the most studied and actively developed electrode materials for Li- and Na-ion batteries. They are related not only to the abundance and non-criticality of manganese but also to their superior stability and high positive electrode potentials.^17,18^ In comparison to oxides, NASICON-structured phosphates containing Mn at the transition metal site offer even higher electrode potentials and a more robust and anionic redox-free framework.^19^ A number of various Mn-containing NASICONs such as Na_3_MnTi(PO_4_)_3_, Na_4_MnV(PO_4_)_3_, and Na_4_MnCr(PO_4_)_3_ were successfully prepared and characterized.^20−24^ However, to the best of our knowledge, a purely Mn-based NASICON phosphate, that is, (Na,Li)_*x*_Mn_2_(PO_4_)_3_, has never been successfully identified.^25^ Maximizing the Mn content in such compounds would potentially bring a number of advantages for positive NIB electrodes such as a higher energy density and structural stability.

At this point, it is instructive to ask if it is possible to prepare purely Mn-based NASICONs at all. If not, can the Mn content be arbitrarily varied, indicative of a solid-solution type behavior or such systems tend to form thermodynamically stable phases with fixed Mn and other transition-metal ratios? Are there any composition limits for the Mn substitution and if this behavior is dependent on the type of co-substituting metal? Answering these questions would significantly improve our understanding and the ability to develop novel NASICON-based electrodes with a high practical value. Despite a substantial research effort, the phase formation and behavior in NASICON systems are only beginning to emerge.^11,26−29^ So far, most of the crystallographic studies of Mn-based NASICON systems indicate random distributions of species at the transition metal site^19,20,24,30^ and, at least, in some cases [Na_3+*x*_Mn_*x*_V_2–*x*_(PO_4_)_3_ (0 ≤ *x* ≤ 1)^31,32^ or Na_3_Mn_*x*_Fe_2–*x*_(PO_4_)_3_ (0 ≤ *x* ≤ 0.4)^33^ a solid-solution type substitution with a seemingly continuously variable Mn content.

In this study, we analyze the phase formation and behavior in the Na_1+2*x*_Mn_*x*_Ti_2–*x*_(PO_4_)_3_ (0.0 ≤ *x* ≤ 1.5) (NMTP) system using a range of experimental and theoretical techniques. Ti(IV) is known to have a structure stabilizing effect in Mn-based spinel oxides^34−36^ and some NASICONs.^37^ Moreover, by utilizing a multi-electron redox behavior [Ti(III) ⇌ Ti(IV); Mn(II) ⇌ Mn(III) ⇌ Mn(IV)], such a system could serve for bifunctional electrodes (as anodes and cathodes simultaneously) in aqueous symmetric batteries.^12^ We believe NMTP to be one of the most suitable model systems for elucidating the general phase formation and thermodynamic behavior, applicable to a wide range of other Mn-based NASICON systems.

We investigate the system by first constructing its composition–temperature phase diagram using the semi-grand canonical Monte Carlo sampling based on an effective cluster expansion (CE) Hamiltonian derived from the density functional theory (DFT) calculations. The system is sampled by covering a complete Mn(II) content range in Na_1+2*x*_Mn_*x*_Ti_2–*x*_(PO_4_)_3_ (namely, *x* = 0.0; 0.25; 0.5; 0.75; 1.0; 1.25, and 1.5) and assuming a maximum occupancy of four sodium atoms per formula unit in the NASICON structure. Afterward, a set of samples corresponding to the same compositions are prepared using two different synthetic routes, namely, the traditional carbon-free solid-state approach and the popular sol–gel technique, yielding a NASICON-carbon composite. The materials structure and phase composition are characterized by powder X-ray diffractometry and complemented by Raman spectroscopy.

## Materials and Methods

### Computational Methodology

The initial input structures for the exploration of compositional and configurational space were taken from the Materials Project database.^38^ Appropriately sized supercells constructed from rhombohedral unit cells containing two Na_1+2*x*_Mn_*x*_Ti_2–*x*_(PO_4_)_3_ formula units (f.u.) were used for representing all studied structures. Supercells with different Mn concentrations (*x* = 0.0; 0.25; 0.5; 0.75; 1.0; 1.25, and 1.5) and the corresponding number of additional Na atoms for charge compensation were used for computational sampling. Due to an exceedingly high number of configurations, we designed a systematic sampling protocol and used it throughout this work. This protocol was based on using a progressively complex computational hierarchy for identifying the lowest energy configurations. At first, for a particular composition and supercell size, all symmetrically non-equivalent configurations are generated and ranked according to their electrostatic Coulomb energy using the Supercell^39^ package. Structures corresponding to the lowest electrostatic energy were taken for further refinement using a pairwise interatomic potential.^40^ These calculations were performed using the General Utility Lattice Program (GULP).^41^ It was immediately noticed that simple electrostatic lattice energy was insufficient in order to sample and locate the lowest energy configurations and that such intermediate refinement by the use of interatomic potentials was essential (Figure S1). Finally, a set of configurations with the lowest and some with the randomly selected energy as found by the interatomic potential screening were selected for evaluation at the DFT level. The standard calculations were carried out using the Vienna Ab Initio Simulation Package (VASP).^42^ An additional refinement of structures laying on the convex hull was carried out by hybrid DFT using the CRYSTAL17 software suite.^43,44^ The full details of these calculations are reported in Supporting Information.

In order to effectively sample the vast compositional-configurational space of this system, a much more effective way to evaluate lattice energies than direct DFT calculations is necessary. CE is a well-established technique for constructing numerically effective models in order to represent a large number of configurations based on a small training set of reference calculations.^45^ CE is a *de facto* standard for computational evaluation of properties in solid-state alloys and systems with random crystalline arrangements. In this work, we use the Clusters Approach to Statistical Mechanics (CASM) code,^46^ which automates the construction and parameterization of effective CE Hamiltonians and implements them in Monte Carlo simulations.^47−49^ Chemical potential-driven Semi-Grand Canonical Monte Carlo (SGCMC) sampling as implemented in CASM was used to construct the composition–temperature phase diagram for the Na_1+2*x*_Mn_*x*_Ti_2–*x*_(PO_4_)_3_ system. Further details on the sampling procedure and the CE approach can be found in Supporting Information.

### Synthesis

A series of Na_1+2*x*_Mn_*x*_Ti_2–*x*_(PO_4_)_3_ systems with different stoichiometries, namely, *x* = 0.0; 0.25; 0.5; 0.75; 1.0; 1.25, and 1.5, were prepared via a conventional solid-sate route by mixing sodium carbonate (Na_2_CO_3_, 99+%, Chempur), manganese (II) carbonate (MnCO_3_, 99+%, Chempur), titanium (IV) dioxide (anatase) (TiO_2_, 99+%, Alfa-Aesar), and ammonium dihydrogen phosphate (NH_4_H_2_PO_4_, 99+%, Chempur) in appropriate ratios. The mixture was ground in 2-propanol using a planetary ball mill for 2 h at 900 rpm. The dried powder was calcined under a flowing nitrogen atmosphere for 8 h at 600 °C and for additional 8 h at 650 °C after intermediate regrinding. The obtained powder was finally processed in 2-propanol using a planetary ball mill for 2 h at 900 rpm in order to achieve a uniform particle size distribution.

A set of samples with identical compositions were also prepared via a popular aqueous sol–gel method by mixing sodium acetate (NaCH_3_COO, 99+%, Chempur), manganese (II) acetate (Mn(CH_3_COO)_2_, 99+%, Chempur), titanium (IV) isopropoxide (Ti(OCH(CH_3_)_2_)_4_, 98+%, Acros), and ammonium dihydrogen phosphate (NH_4_H_2_PO_4_, 99+%, Chempur) in appropriate ratios. Citric acid as a complexing agent was added to the solution at a 2:1 acid and transition metal ratio. The water from solutions was evaporated by stirring on a hot plate, followed by overnight drying in a vacuum oven at 100 °C. The prepared xerogels were calcined under a flowing nitrogen atmosphere for 4 h at 400 °C and then for additional 8 h at 650 °C after intermediate regrinding. The obtained powder was also finally processed in a planetary ball mill for 2 h at 900 rpm in order to achieve a uniform particle size distribution.

### Structural Characterization

All powder X-ray diffraction (XRD) data were obtained on a Rigaku SmartLab X-ray diffractometer equipped with a graphite monochromator and point detector using Cu Kα_1,2_ radiation. The XRD patterns were obtained with a 0.01° step size at a 1°/min scan rate. The peak line shape was calibrated by recording a pattern of the NIST SRM 660c standard under the same conditions as analyzed samples. Rietveld refinements of XRD patterns were performed using the GSAS-II software suite.^50^ The theoretically predicted lowest energy Na_3_MnTi(PO_4_)_3_ structure on the convex hull was used as an initial input and gave the best quality fit in the Rietveld refinement procedure.

### Spectroscopic Characterization

Raman spectra were recorded at room temperature using combined Raman spectroscopy and scanning near-field optical microscopy (SNOM) (WiTec Alpha 300 R) equipped with a 532 nm laser excitation source. For the measurements, a 600 g/mm grating was used together with 50× objective for the backscattering signal collection. The signal acquisition time was set to 20 s, and the laser power was set to 2.25 mW. All spectra were corrected by background subtraction and normalization of peak intensities by the area integral.

## Results and Discussion

### Computational Construction of the NMTP Composition–Temperature Phase Diagram

**Figure 1:**
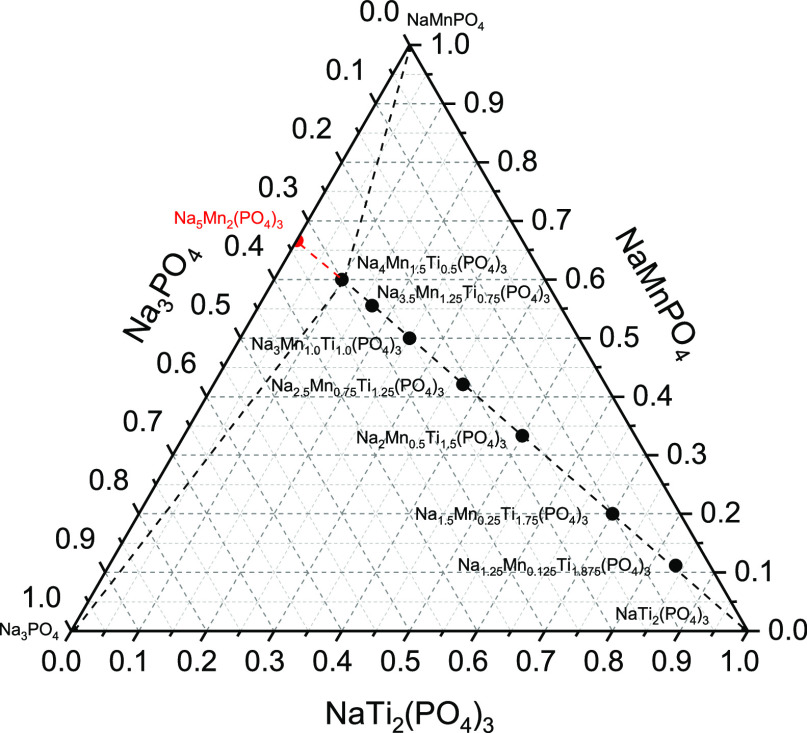
Ternary NMTP phase diagram with NaTi_2_(PO_4_)_3_, NaMnPO_4_, and Na_3_PO_4_ as pseudo end members. The dashed composition line corresponds to the Na_1+2*x*_Mn_*x*_Ti_2–*x*_(PO_4_)_3_ system. The black dots mark compositions studied in this work, and the hypothetical Na_5_Mn_2_(PO_4_)_3_ phase is denoted by a red dot.

There are several ways of analyzing the thermodynamic stability and phase formation in NASICON-structured systems. One could construct the phase diagram in terms of pure elements, binary oxides, or phosphates. In this work, the latter was chosen and NaTi_2_(PO_4_)_3_ is treated as a parent compound. The aliovalent substitution of Ti(IV) with Mn(II) requires two additional Na(I) for charge compensation. In this way, NaTi_2_(PO_4_)_3_, NaMnPO_4_, and Na_3_PO_4_ are considered as “pseudo end members” in a ternary phase diagram (Figure 1), and the process of mixing in this system is then described as
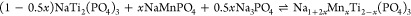
1Therefore, the NASICON-structured Na_1+2*x*_Mn_*x*_Ti_2–*x*_(PO_4_)_3_ system can be considered as lying on a composition line connecting NaTi_2_(PO_4_)_3_ and hypothetical Na_5_Mn_2_(PO_4_)_3_ (see Figure 1). However, in this work, we assume manganese to be available only in a divalent state and the maximum of four available sodium sites per NASICON formula unit.^51^ Hence, the Na_4_Mn_1.5_Ti_0.5_(PO_4_)_3_ composition represents a physical end point for possible NASICON compositions in NMTP (Figure 1).

**Figure 2:**
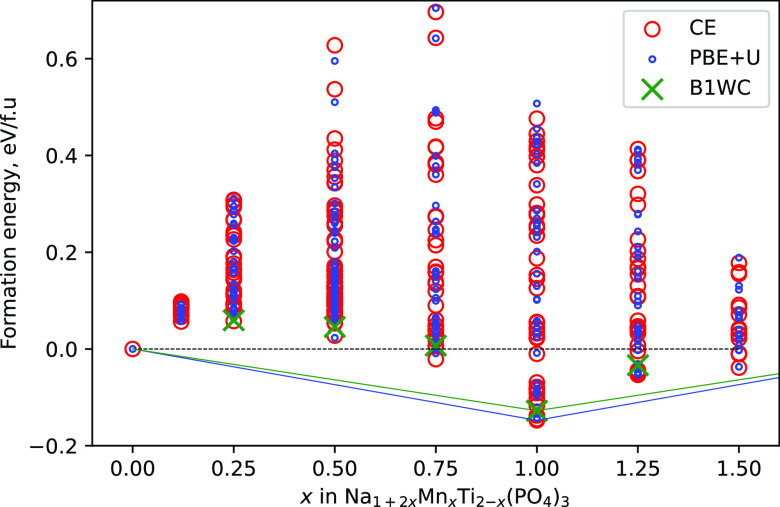
Formation energies of Na_1+2*x*_Mn_*x*_Ti_2–*x*_(PO_4_)_3_ configurations depending on *x*. Blue circles correspond to the formation energies obtained at the PBE + *U* level of theory. Green crosses mark formation energies refined using the hybrid B1WC functional. Red circles denote formation energies calculated by the CE model developed in this work. The convex hull obtained from PBE + *U* is marked by a blue line, while the green line corresponds to the hybrid DFT refined convex hull.

A set of fixed discrete Ti/Mn ratios spanning the entire composition range between NaTi_2_(PO_4_)_3_ and Na_4_Mn_1.5_Ti_0.5_(PO_4_)_3_ in equal intervals was chosen in order to analyze the energetics and construct the phase diagram for this system. The fixed *x* values were set to 0.0, 0.25, 0.5, 0.75, 1.0, 1.25, and 1.5. At each composition, the configurational space corresponding to various possible arrangements and orderings of Na/V_Na_ and Mn/Ti was sampled separately. The protocol is described in detail in the Materials and Methods section and Supporting Information. A high quality CE (CE) Hamiltonian was constructed using the complete information covering all compositions and configurations. Additional configurations were also added to the model by an iterative refinement using a Monte Carlo search. If a new lower CE energy configuration was discovered, it was fed back into the CE fit and a new Monte Carlo search was performed. This was repeated multiple times until no new configurations with lower energy were identified. Figure 2 presents the sampling results and energy evaluations at different levels of theory (PBE + U, hybrid B1WC, and CE). Each data point on the plot corresponds to a formation energy of a single NMTP configuration with respect to pseudo end member energies. One can immediately notice a very good agreement between different theoretical approaches, especially for the most stable configurations laying on the envelope formed around the lowest energy structures, also known as the convex hull (blue line in Figure 2). The results strongly suggest that the quality of our developed CE model is sufficiently accurate for describing the principal energetic and thermodynamic properties of the NMTP system and make useful predictions for further understanding and experimental guidance.

Obviously, most of the sampled configurations, regardless of composition, have positive formation energies with respect to the end phases at 0 K (Figure 2). Nevertheless, few configurations at *x* = 0.75, 1.25, and 1.50 show some stability, as manifested by relatively small but still negative formation energies in the range of ∼10 to 50 meV/f.u. However, the composition corresponding to *x* = 1.0 (Na_3_MnTi(PO_4_)_3_) is markedly (∼180 meV/f.u.) more stable than others. Moreover, it is evident that only Na_3_MnTi(PO_4_)_3_ lays directly on the convex hull of this system, whereas all other compositions even with negative formation energies are above it. These results suggest that at 0 K, the NMTP system will tend to decompose into Na_3_MnTi(PO_4_)_3_ and NaTi_2_(PO_4_)_3_ for *x* < 1.0 and into Na_3_MnTi(PO_4_)_3_ and NaMnPO_4_ for *x* > 1.0.

**Figure 3:**
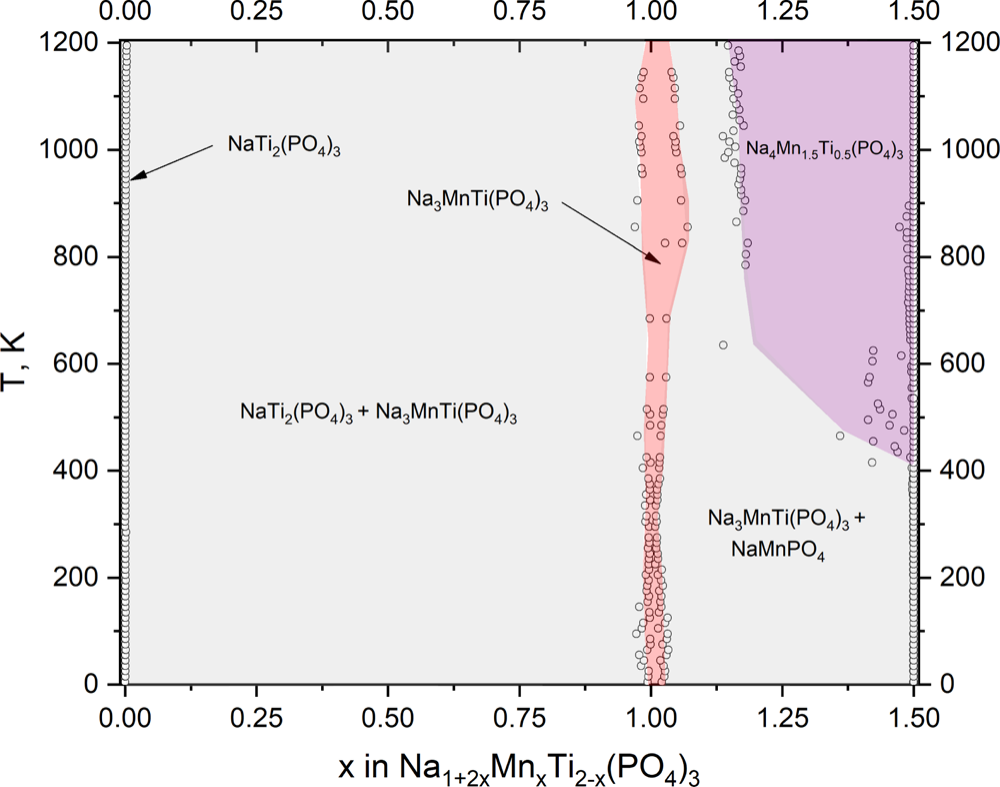
Semi-grand canonical Monte Carlo calculated composition–temperature phase diagram for the Na_1+2*x*_Mn_*x*_Ti_2–*x*_(PO_4_)_3_ (0.0 ≤ *x* ≤ 1.5) system. Empty circles originate from the numerical integration of chemical potential versus composition (see Supporting Information).

The finite temperature phase diagram was obtained by semi-grand canonical Monte Carlo simulations using the CE Hamiltonian. The phase stability boundaries, presented in Figure 3, are identified by numerically analyzing the discontinuities in composition with respect to chemical potential. The composition was sampled by varying the Mn and Na content simultaneously in order to preserve electroneutrality. A very similar approach was recently successfully demonstrated for the Na_1+*x*_Zr_2_Si_*x*_P_3–*x*_O_12_ NASICON system.^26^ Our constructed temperature–composition diagram indicates a well-pronounced stability of the Na_3_MnTi(PO_4_)_3_ phase at all studied temperatures between 0 and 1200 K. Therefore, all compositions for *x* < 1.0 correspond to a biphasic co-existence of Na_3_MnTi(PO_4_)_3_ and NaTi_2_(PO_4_)_3_ at all studied temperatures. On the Mn-rich side for *x* > 1.0, the results indicate a biphasic co-existence of Na_3_MnTi(PO_4_)_3_ and NaMnPO_4_ at *T* < 400 K, whereas at a higher temperature, there is some visible stability of Na_4_Mn_1.5_Ti_0.5_(PO_4_)_3_. However, the latter might be an artifact of our modelling framework due to the inability to include either Na_5_Mn_2_(PO_4_)_3_ or NaMnPO_4_ into the SGCMC protocol. Hence, Na_4_Mn_1.5_Ti_0.5_(PO_4_)_3_ simply corresponds to the natural end point of the available composition window for this system. It is possible that this might provide some artificial but not truly physical stability for this composition in this model.

Another interesting feature one could observe in this phase diagram is a finite *x* range of stability around *x* ≈ 1.0 for all studied temperatures (Figure 3). This also might be an artifact of numerical differentiation carried out during modelling of this diagram or indicate a true finite stability range of this composition as Na_3+2δ_Mn_1+δ_Ti_1-δ_(PO_4_)_3_ with δ ≈ 0.03–0.05. In order to investigate these features in more detail and test our theoretical predictions, we decided to carry out a number of experiments, the results of which are presented in the following sections.

### Synthesis and Structural Analysis

A number of recent reports demonstrate successful syntheses of single-phase Na_1+2*x*_Mn_*x*_Ti_2–*x*_(PO_4_)_3_ with *x* = 0.5, 1.0, and 1.2.^12,19,52−54^ All these studies employed a simple aqueous sol–gel method, which typically involves mixing metal acetates, alkoxides, and NH_4_H_2_PO_4_ together with a complexing agent (e.g., citric acid) for the preparation of precursor gels. The calcination of these gels in an inert atmosphere at 600–650 °C yields single-phase NMTP compounds purposely embedded in a conductive carbon matrix for electrochemical characterization.^12,19,52,53^ The phase composition and structure of the obtained samples are conventionally characterized by powder XRD. However, such XRD patterns typically show significant diffraction peak broadening and an elevated background. The obvious reasons for this effect are a small particle size, an inferior degree of crystallinity resulting from a significant presence of amorphous phases, and X-ray scattering by the carbon matrix. These features make the XRD patterns difficult to interpret, leading to inconclusive results regarding the phase composition and crystalline structure of such systems.

In this work, a number of samples corresponding to fixed stoichiometries of Na_1+2*x*_Mn_*x*_Ti_2–*x*_(PO_4_)_3_, with *x* = 0.0, 0.25, 0.5, 0.75, 1.0, 1.25, and 1.5, were prepared using a popular aqueous sol–gel technique as well as traditional solid-state synthesis assisted by mechanical ball milling. Solid-state synthesis allows the preparation of carbon-free samples with a higher degree of crystallinity and a larger primary particle size. Indeed, in this work, the NMTP samples obtained by this approach showed significantly reduced XRD peak broadening and background level compared to those prepared by the sol–gel method. This enables us to identify the phase composition of these samples much more precisely and subsequently perform Rietveld structure refinement at a much better quality than would be possible in sol–gel prepared NMTP/carbon composites.

**Figure 4:**
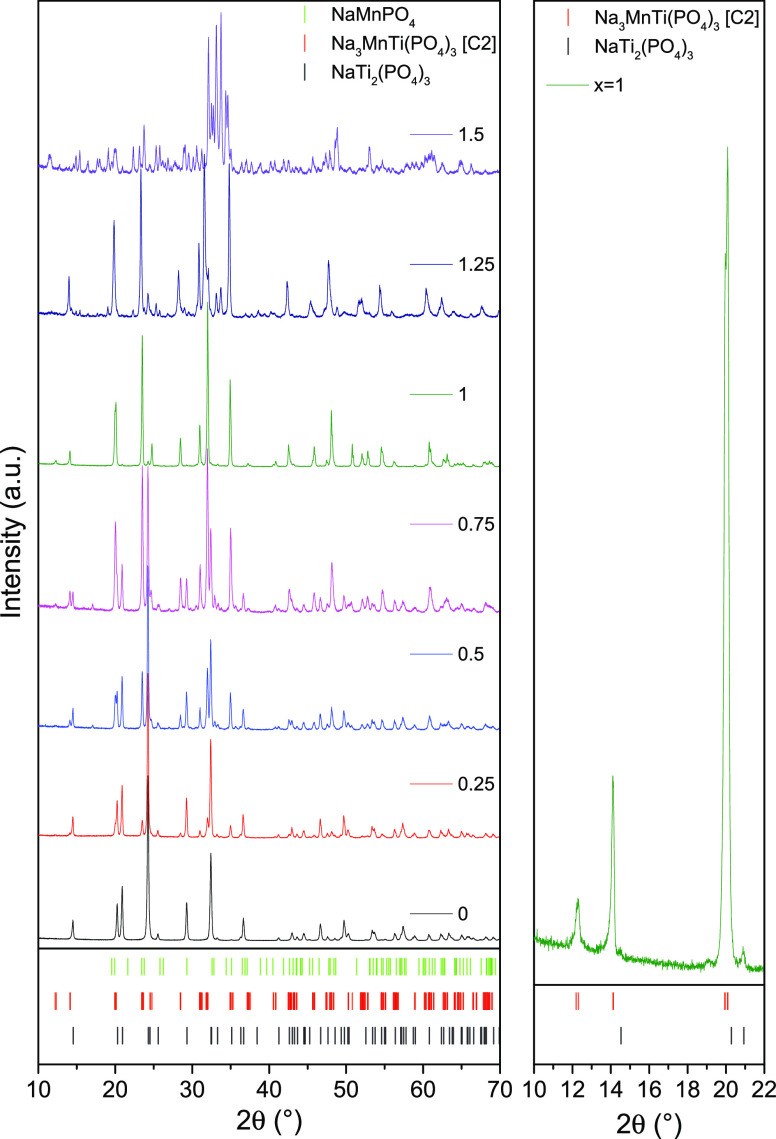
Powder XRD patterns of Na_1+2*x*_Mn_*x*_Ti_2–*x*_(PO_4_)_3_ (0.0 ≤ *x* ≤ 1.5) series prepared by a solid-state synthesis route. The inset on the right shows the superstructure peak at 2θ ≈ 12.2°.

The obtained powder XRD patterns of Na_1+2*x*_Mn_*x*_Ti_2–*x*_(PO_4_)_3_ series at specified compositions are presented in Figure 4. The results indicate that in the *x* range from 0.0 to 1.0, NMTP obviously crystallizes in NASICON-type structure. In agreement to previous reports, our results show its characteristic XRD pattern and confirm the existence of a distinct Na_3_MnTi(PO_4_)_3_ phase for *x* = 1.0 (Figure 4). One of the features distinguishing the XRD pattern of Na_3_MnTi(PO_4_)_3_ from NaTi_2_(PO_4_)_3_ is a small superstructure peak at 2θ ≈ 12.2° which is usually related to sodium ordering, accompanied by rhombohedral-to-monoclinic transition. In line with the existing nomenclature on NASICONs, we will be referring to this phase as α-Na_3_MnTi(PO_4_)_3_ further on. The XRD patterns for intermediate Ti/Mn ratios of *x* ranging from 0.25 to 0.75 unambiguously demonstrate that Ti substitution with Mn in NMTP does not show a solid-solution type behavior with continuously varying Ti/Mn ratio but, as predicted theoretically, is indicative of a phase separation into NaTi_2_(PO_4_)_3_ and Na_3_MnTi(PO_4_)_3_ phases. The superstructure reflection at 2θ ≈ 12.2° is still slightly visible in compositions with *x* down to 0.5, confirming the presence of α-Na_3_MnTi(PO_4_)_3_ and the fact that it remains in the same symmetry regardless of sample composition. The disappearance of this feature at *x* = 0.25 is probably due to the relatively low amount of the α-Na_3_MnTi(PO_4_)_3_ phase in a sample. Quantitative analysis of XRD patterns also supports this view because the fraction of the NaTi_2_(PO_4_)_3_ phase in this range varies approximately linearly with Mn content (Table 1).

**Table 1:** Structural Parameters Obtained by Rietveld Refinement of Powder XRD Patterns for Na_1+2*x*_Mn_*x*_Ti_2–*x*_(PO_4_)_3_ (0.25 ≤ *x* ≤ 1.0) Series Prepared by Solid-State Synthesis^a^

	*R*3̅*c*	*C*2			
space group	*x* = 1.0	*x* = 1.0	*x* = 0.75	*x* = 0.5	*x* = 0.25
*a*, Å	8.82944(11)	15.2567(30)	15.273(13)	15.270(16)	15.253(15)
*b*, Å	8.82944(11)	8.89520(20)	8.8726(8)	8.8758(10)	8.8750(10)
*c*, Å	8.82944(11)	8.8314(11)	8.840(5)	8.842(6)	8.852(5)
β, deg	60.4592(14)	124.8061(28)	125.033(12)	124.955(14)	124.957(14)
unit cell volume, Å^3^	491.7795	984.086	980.96	982.23	982.04
NaTi_2_(PO_4_)_3_ fraction, % w/w	1.65	1.35	33.04	58.69	82.10
*R*_wp_, %	3.19	9.52	12.82	11.54	10.37
GOF	2.28	1.65	1.66	1.50	1.39
χ_red_^2^	5.19	2.71	2.75	2.25	1.92

a Note that the reported unit cell parameters are expressed in rhombohedral representation.

The presence of α-Na_3_MnTi(PO_4_)_3_ might still be detected at *x* = 1.25, but other impurity phases such as orthorhombic NaMnPO_4_ also start to appear. Nevertheless, the superstructure peak almost disappears in this *x* range. It is also important to note that in the *x* range above 1.0, the main peaks corresponding to α-Na_3_MnTi(PO_4_)_3_ are slightly shifted toward lower 2θ values (e.g., 31.57° vs 32.01°). This might indicate a finite Mn solubility range in NMTP with *x* = 1.0 + δ and the existence of a slightly Mn-rich α-Na_3+2δ_Mn_1+δ_Ti_1-δ_(PO_4_)_3_ phase. However, unlike previously reported by Liu et al.,^53^ this range must be relatively narrow. Finally, the powder XRD patterns for the *x* = 1.5 composition no longer indicate any presence of NASICON-structured phases. Only orthorhombic NaMnPO_4_ was unambiguously identified in the data, and the rest of the pattern was too complex for attributing any remaining phases. These results are in excellent agreement with our theoretical predictions. Our SGCMC simulated phase diagram not only indicates the existence of a stable Na_3_MnTi(PO_4_)_3_ phase at *x* = 1.0 and its biphasic coexistence with NaTi_2_(PO_4_)_3_ for *x* < 1.0 and NaMnPO_4_ for *x* > 1.0 but also suggests a finite Mn solubility range around *x* = 1.0 + δ (Figure 3).

**Figure 5:**
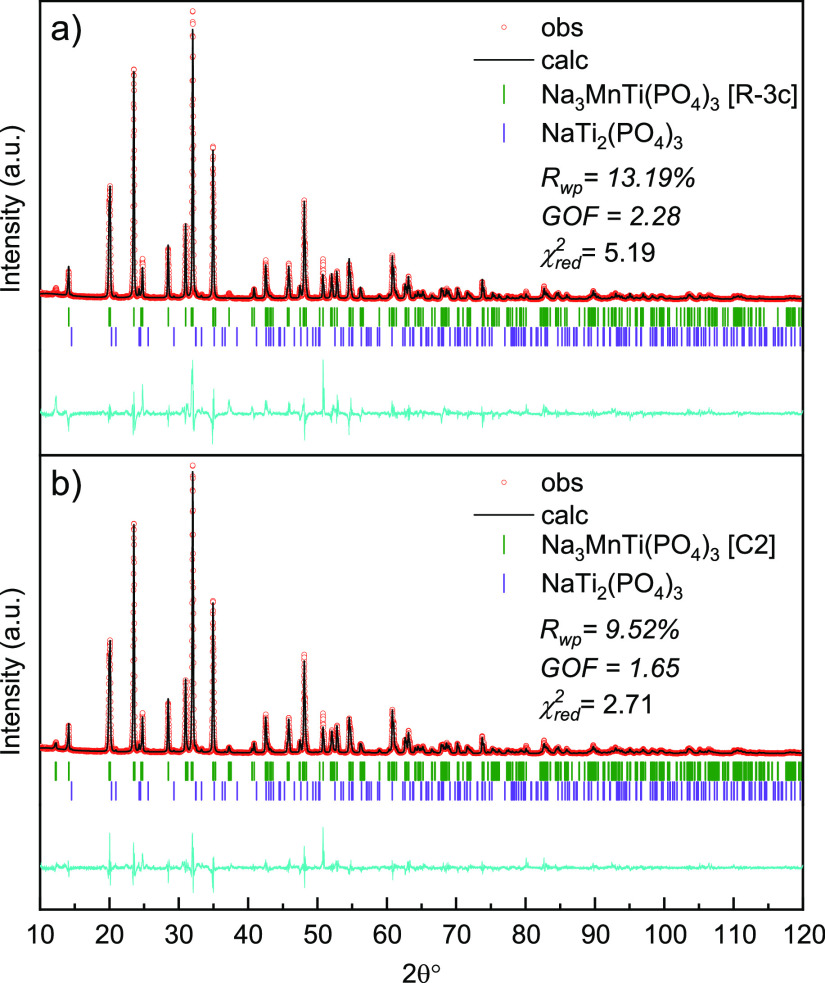
Results of Rietveld refinement of the powder XRD pattern of Na_3_MnTi(PO_4_)_3_ (*x* = 1.0) using (a) *R*3̅*c* and (b) *C*2 space groups.

The crystal structure of α-Na_3_MnTi(PO_4_)_3_ was refined by the Rietveld method. All the previous studies so far attributed the high-symmetry *R*3̅*c* (no. 167) space group to Na_3_MnTi(PO_4_)_3_.^12,19,52,53^ Therefore, at first, we also tried to refine the structure in this space group. Although the refinement shows reasonable agreement with the experimental data, the reflection at ∼12.2° is completely missing (Figure 5a). The latter peak is usually attributed to a superstructure due to sodium ordering and rhombohedral-to-monoclinic transition in NASICON-structured systems with 3 Na atoms per formula unit (e.g., Na_3_V_2_(PO_4_)_3_, Na_3_Ti_2_(PO_4_)_3_, Na_3_Fe_2_(PO_4_)_3_, Na_3_Al_2_(PO_4_)_3_, Na_3_Cr_2_(PO_4_)_3_, etc.), especially at lower temperatures.^55−58^ To the best of our knowledge, such distortion has not been reported in the Na_3_MnTi(PO_4_)_3_ system yet. However, considering the low intensity of this reflection and the usually applied preparation methods (e.g., sol–gel synthesis), it is likely that this peak was invisible due to low sample crystallinity and a high carbon content. Therefore, in the next attempt, we tried to refine the obtained pattern using the most common space groups such as *C*2/*c* (no. 15), *C*2 (no. 5), and *P*1̅ (no. 2).^56,59^ For this purpose, the lowest energy configuration from the DFT/CE sampling corresponding to *x* = 1.0 and identified as belonging to the monoclinic *C*2 space group gave a very good initial guess and rapidly converged to the final solution, with the best overall *R*_wp_ = 9.52%, a goodness of fit (GOF) of 1.65, and χ_red_^2^ = 2.71. In comparison, for *C*2/*c*, the results were *R*_wp_ = 11.8%, GOF = 2.04, and χ_red_^2^ = 4.16 and for *P*1̅ *R*_wp_ = 11.297%, GOF = 1.96 and χ_red_^2^ = 3.83. The results of Rietveld refinement are presented in Figures 5 and S9 and Tables 1 and S2.

The Rietveld refinement for the *x* range between 0.25 and 0.75, where NaTi_2_(PO_4_)_3_ is identified as the only impurity phase, revealed a minor variation in lattice parameters and volume which might probably be attributed to numerical variation in the fitting algorithm (Table 1). For *x* = 1.25, a reliable refinement was not achieved, probably due to the presence of additional impurity phases which we were not able to identify with a sufficient degree of certainty. Nevertheless, the observed slight peak shift toward lower 2θ indicates a possible small expansion of the unit cell, confirming that a narrow solid solubility range might exist around *x* = 1.0 + δ in Na_1+2*x*_Mn_*x*_Ti_2–*x*_(PO_4_)_3_. The XRD analysis is also unable to provide information on the Mn/Ti ordering in NMTP due to the similarity in their X-ray atomic form factors. Therefore, we assume a random distribution of transitions metals in the NASICON structure in this work (Figure S2).

For a comparative purpose, the Na_1+2*x*_Mn_*x*_Ti_2–*x*_(PO_4_)_3_ series for 0.25 ≤ *x* ≤ 1 was also prepared using a sol–gel method. The obtained XRD patterns confirm the presence of a single Na_3_MnTi(PO_4_)_3_ phase at *x* = 1.0 but no superstructure peak at low 2θ (Figure S10). In the case of *x* < 1.0, the diffraction peaks appear to be slightly shifted toward larger 2θ. However, the appearance of significant broadening and asymmetry of the peaks suggest that this seeming shift could be a result of peak overlap between Na_3_MnTi(PO_4_)_3_ and NaTi_2_(PO_4_)_3_ phases rather than a change in lattice parameters. This hypothesis is confirmed by an additional experiment, where the carbonaceous phase for a sample with *x* = 0.5 was carefully removed by heating the powder in a flowing air atmosphere. The obtained XRD pattern is shown to be almost identical to the solid-state synthesized sample (Figure S11).

### Raman Spectroscopic Analysis of Phase Composition

**Figure 6:**
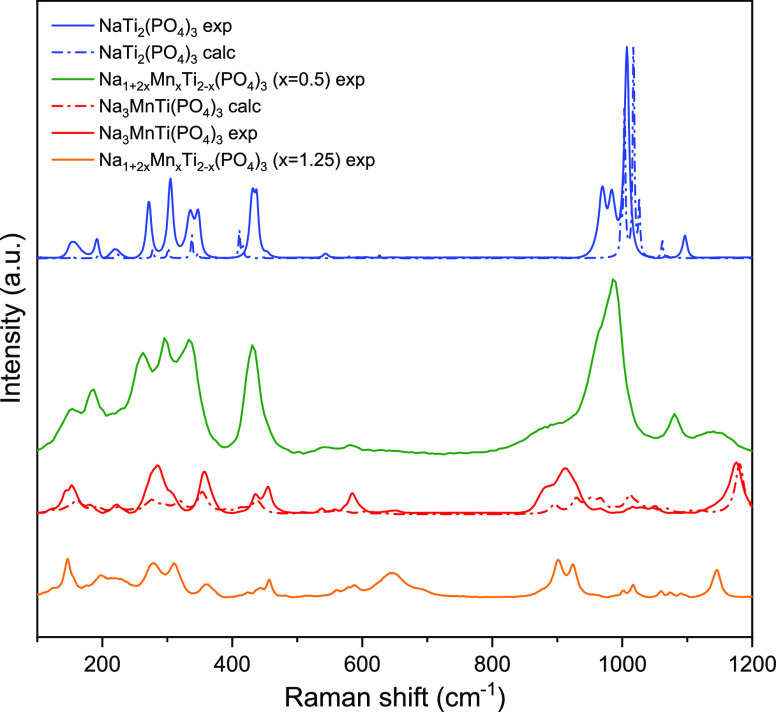
Comparison of experimental and periodic hybrid DFT-calculated Raman spectra for NaTi_2_(PO_4_)_3_ (blue), Na2Mn_0.5_Ti_1.5_(PO_4_)_3_ (green), α-Na_3_MnTi(PO_4_)_3_ (red), and Na_3.5_Mn_1.25_Ti_0.75_(PO_4_)_3_ (orange).

In order to analyze the phase formation and composition of the NMTP system, we also employed Raman spectroscopy. Raman spectroscopy is a non-destructive tool suitable for phase identification and fingerprinting. It is more sensitive to local order and composition than XRD, making it a useful complementary technique to confirm our results regarding the biphasic behavior of the NMTP system. Experimental Raman spectra were recorded for Na_1+2*x*_Mn_*x*_Ti_2–*x*_(PO_4_)_3_ samples corresponding to *x* = 0.0, 0.5, and 1.0. In addition, we also calculated Raman spectra by periodic hybrid DFT using the CRYSTAL17 code for *x* = 0.0 and 1.0. The obtained experimental and computational results are presented in Figure 6. One can immediately see that Raman spectroscopy is not only able to distinguish between NaTi_2_(PO_4_)_3_ and α-Na_3_MnTi(PO_4_)_3_ but also even much more sensitive to the local structure than XRD. Highly symmetric NaTi_2_(PO_4_)_3_ shows much sharper and less overlapping peaks than low-symmetry α-Na_3_MnTi(PO_4_)_3_. The presented experimental Raman spectrum of NaTi_2_(PO_4_)_3_ is in agreement with those reported in previous studies and our calculations.^60,61^ Even though the peaks are slightly shifted in the calculated spectrum, the main bands are in very good agreement with experiments. High-intensity bands associated with PO_4_ symmetric (ν_1_) and asymmetric (ν_3_) stretching vibrations can be observed in the 930–1120 cm^–1^ range, whereas low-intensity asymmetric (ν_4_) and high-intensity symmetric (ν_2_) bending vibrations are visible at 545 cm^–1^ and 430 cm^–1^, respectively. Bands observed in the 350–330 cm^–1^ range can be associated to Ti–O bond vibrations, and bands below 250 cm^–1^ are attributed to lattice vibrations.^61^ The positions and intensities of some bands in the spectrum of α-Na_3_MnTi(PO_4_)_3_ are different from those in NaTi_2_(PO_4_)_3_. However, due to the less symmetric environment, the main PO_4_ modes split and significantly overlap, reducing the main peak intensity. ν_1_ (PO_4_) stretching vibrations are shifted to lower frequencies (850–980 cm^–1^), whereas ν_3_ are shifted to higher frequencies (1180 cm^–1^). Moreover, some additional bands appear in 550–700 cm^–1^ in both measured and calculated spectra, possibly resulting from lowered symmetry and activation of additional Ti–O bands which are typically Raman-inactive in highly symmetric NaTi_2_(PO_4_)_3_.^62^ Detailed analysis of calculated spectra shows that these bands are due to P–O and P–O–Na with some contribution of Ti–O vibrations. Notice that there is a stretching Mn–O bond vibration at 296 cm^–1^. However, the main contributions of Mn appear at even lower frequencies.

In good agreement with our computational and XRD results, Raman spectroscopy also strongly suggests that *x* = 0.5 is a mixture of two phases. The observed spectrum for this sample looks like a combination of *x* = 0.0 and 1.0 spectra; however, the NaTi_2_(PO_4_)_3_ bands are much higher than α-Na_3_MnTi(PO_4_)_3_. Nevertheless, the agreement between DFT-calculated and recorded spectra is very good in for the pure phases.

### Crystal Orbital Overlap Population Analysis of Chemical Bonding

One of the remaining key questions in the field of NASICON materials is what are the key driving forces and interactions responsible for their thermodynamic stability as well as electrochemical performance. Most of the previous studies identified sodium sublattice ordering in NASICONs as one of the key factors governing the phase formation and stability.^26,28^ Most of the known NASICON electrode materials such as Na_x_Ti_2_(PO_4_)_3_, Na_x_V_2_(PO_4_)_3_, Na_x_Fe_2_(PO_4_)_3_, Na_x_Cr_2_(PO_4_)_3_, and Na_x_FeV(PO_4_)_3_ tend to crystallize in a high-symmetry rhombohedral structure for *x* = 1.0 and 4.0 but distort to lower-symmetry monoclinic structures at *x* = 3.0, especially at low temperatures. Most of these transition metals show very weak or no Jahn–Teller distortion, which is also the case for high-spin Mn(II) in octahedral coordination. Therefore, it is unlikely that Jahn–Teller effects are important for phase formation in this NMTP system either. In order to identify other possible causes for instability of Mn-rich compositions in NMTP besides sodium ordering, we performed a number of electronic structure analyses. At first, the Bader atomic charges were calculated for all atoms and correlated with respect to *x* and formation energy for all configurations. The complete set of results is presented in Figure S7. One can see that there is certainly some charge redistribution taking place with respect to system composition in Na_1+2*x*_Mn_*x*_Ti_2–*x*_(PO_4_)_3_. Mn, Ti, and Na at M2 position atomic charges all show a decreasing trend with increasing Mn content. Only the Na at M1 positions of the NASICON structure show increasing atomic charge with respect to *x*. This supports the idea that sodium ordering and the sublattice structure have an effect on the phase energetics in this system. In addition, we also evaluated the bond lengths and octahedral distortions around the transition metals. The results in Figure S8 show that the most stable structures, namely, NaTi_2_(PO_4_)_3_ and α-Na_3_MnTi(PO_4_)_3_, show the shortest Ti–O bond lengths and the weakest [TiO_6_] octahedral distortion in the compositional-configurational space. On the other hand, α-Na_3_MnTi(PO_4_)_3_ shows the longest Mn–O bond length and some of the strongest [MnO_6_] octahedral distortion. This suggests that there might be some unfavorable interactions between Mn and O with a potential limit on how much Mn might be allowed in the NASICON-type structure.

**Figure 7:**
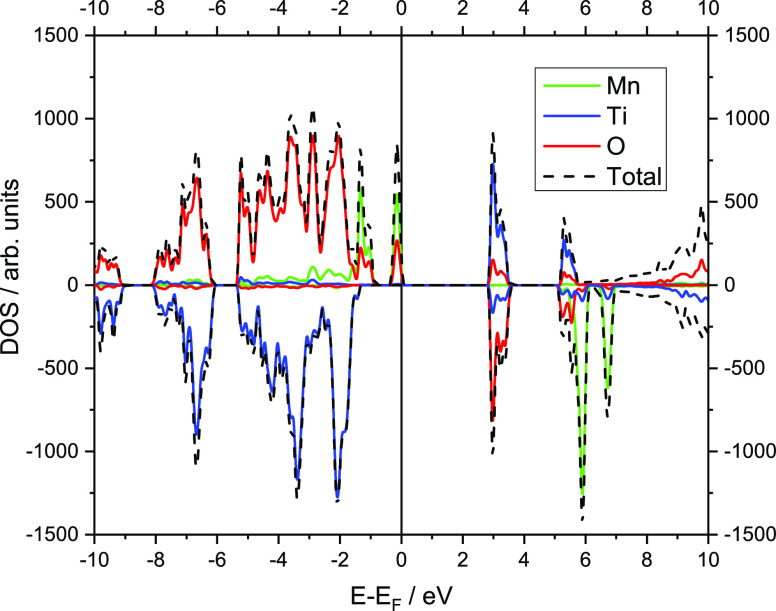
Total and partial DOS calculated with the B1WC functional for α-Na_3_MnTi(PO_4_)_3_. The top of the filled states is taken as zero. Negative values represent spin-down electrons; (black dashed line) total DOS, (red solid line) O, (blue solid line) Ti, and (green solid line) Mn.

**Figure 8:**
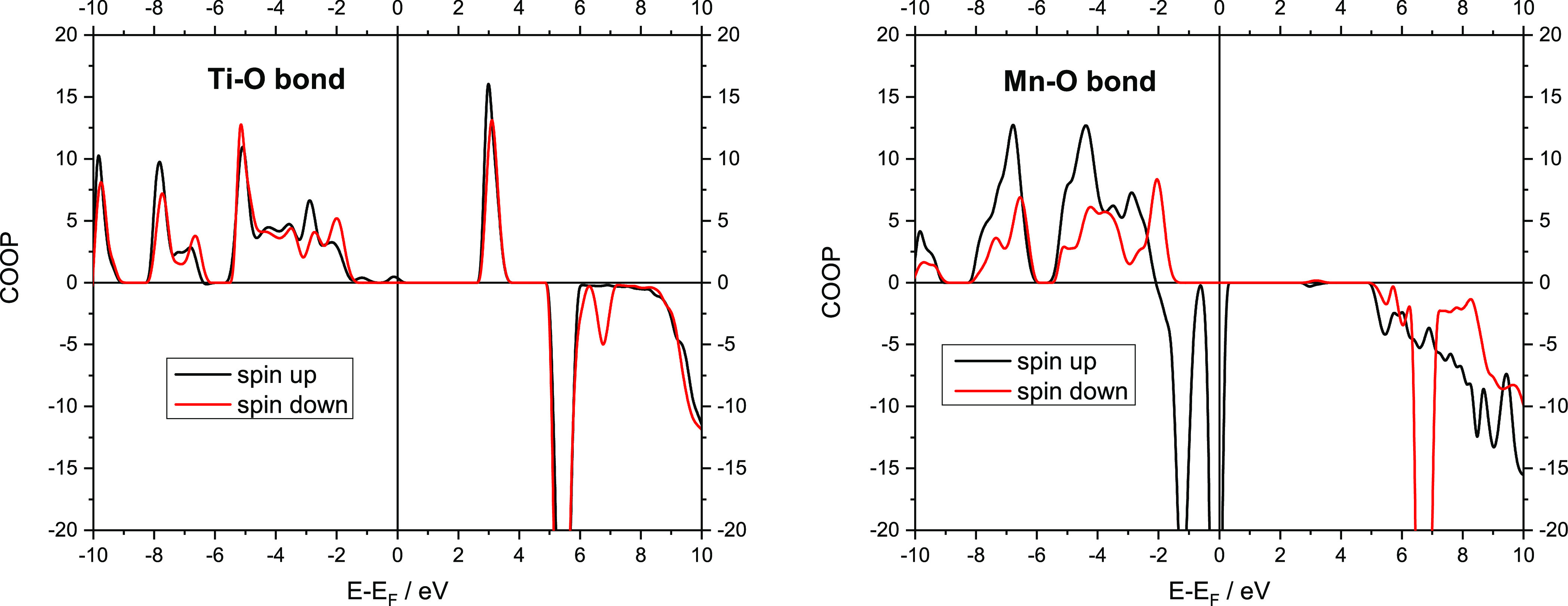
COOP in arbitrary units for (left) the Ti–O bond and (right) Mn–O bond in α-Na_3_MnTi(PO_4_)_3_. Positive COOP values correspond to bonding states, and negative COOP values correspond to anti-bonding states; (black line) spin-up states and (red line) spin-down states.

Therefore, we decided to analyze the electronic structure of the Mn–O bond in terms of total/partial density of states (DOS) and crystal orbital overlap populations (COOPs) and compare it to the Ti–O bond. COOP is a valuable descriptor of chemical bonding which is obtained from an overlap population-weighted DOS.^63^ Positive COOP values correspond to overlap and indicate bonding, zero values correspond to non-bonding, and negative values correspond to anti-bonding interactions. The calculated total and atom projected DOS for α-Na_3_MnTi(PO_4_)_3_ are presented in Figure 7. One can immediately notice a significant difference between the Mn–O and Ti–O bonds. As was also already pointed out in our previous study, there is very little hybridization between Ti 3d states and O 2p states, resulting in more ionic bonds and a wide band gap (∼4 eV) in NaTi_2_(PO_4_)_3_.^51^ On the other hand, the Mn–O bond has a completely different chemical character. There is a much stronger hybridization of Mn 3d states and O 2p states as indicated by the presence of filled Mn and O states close to the Fermi energy in Figure 7. It is instructive to ask what is the bonding character of these states. The comparison of COOP for Ti–O and Mn–O bonds in α-Na_3_MnTi(PO_4_)_3_ is presented in Figure 8. As a strong bond with more ionic character, Ti–O shows little positive values close to *E*_f_ and no anti-bonding character up to energies much higher than *E*_f_. On the other hand, the presence of filled anti-bonding spin-up states for the Mn–O bond just below the Fermi level indicates that this bond is significantly less stable than Ti–O. We believe that the inherent instability of the Mn–O bond is another factor governing not only the phase stability and formation in Mn-based NASICON systems but also their electrochemical stability.

## Conclusions

In this study, we investigated a NASICON-structured Na_1+2*x*_Mn_*x*_Ti_2–*x*_(PO_4_)_3_ (0.0 ≤ *x* ≤ 1.5) system, which is a useful Na-ion battery electrode active material. The peculiarities of phase formation and thermodynamic stability are analyzed by constructing a composition–temperature phase diagram based on extensive computational sampling using DFT, CE, and semi-grand canonical Monte Carlo methods. The analysis indicates that this system does not show a solid-solution type behavior across different Mn-contents *x* but rather phase-separates into stoichiometric Na_3_MnTi(PO_4_)_3_ and NaTi_2_(PO_4_)_3_ for *x* < 1.0 or into Na_3_MnTi(PO_4_)_3_ and NaMnPO_4_ for *x* > 1.0 at all studied temperatures. Our computational predictions are supported by experimental findings obtained using X-ray diffractommetry and Raman spectroscopy on solid-state and sol–gel prepared samples. The experiments strongly suggest that phase separation is driven by the formation of thermodynamically more stable sodium ordered monoclinic α-Na_3_MnTi(PO_4_)_3_ with space group *C*2. The crystal structure of the latter phase was refined using the Rietveld method. In addition, to sodium ordering which is identified as one of the main driving forces behind the phase formation and stability, the chemical character of the Mn–O bond as compared to Ti–O is also suggested as another important factor. The DOSs and COOP analysis show that in addition to much stronger hybridization between the transition metal 3d states and oxygen 2p states in the Mn–O bond, its anti-bonding character is also much stronger, resulting in a weaker bond compared to the Ti–O bond. We believe that these results will not only clarify some important questions regarding the thermodynamic properties of Mn-based NASICON compounds but also provide helpful guidance for a more general understanding of other Na and Li polyanionic electrode systems.

## Supporting Information Available

Detailed description of computational (DFT, CE, SGMC) methods and protocols and additional experimental XRD details and results (PDF)
Powder XRD solved crystal structure of the Na ordered α-Na_3_MnTi(PO_4_)_3_ phase (CIF)

## Supplementary Material

cm1c02775_si_001.pdf

cm1c02775_si_002.cif
