# Supplementary material for: Peculiarities of Phase Formation in Mn-Based Na SuperIonic Conductor (NaSICon) Systems: The Case of Na1+2xMnxTi2–x(PO4)3 (0.0 ≤ x ≤ 1.5)
Source: Chem Mater. 2021 Oct 21;33(21):8394–403. doi: 10.1021/acs.chemmater.1c02775 (PMC8721591; doi:10.1021/acs.chemmater.1c02775)
Supplement: Supplementary file 1 — cm1c02775_si_001.pdf [file cm1c02775_si_001.pdf]

Supporting Information for Peculiarities of Phase Formation in Mn-Based Na SuperIonic Conductor (NaSICon) Systems: The Case of $Na_{1+2x}Mn_xTi_{2-x}(PO_4)_3$ ($0.0 \leq x \leq 1.5$)

Gustautas Snarskis, Jurgis Pilipavičius, Denis Gryaznov, Lina Mikoliūnaitė, and Linas Vilčiauskas*

E-mail: linas.vilciauskas@ftmc.lt

Computational Details

Configurational sampling

For example, at $x = 0.5$ there are 1770 symmetrically non-equivalent configurations having different Na/V_{Na} and Ti/Mn arrangements within the 2x1x1 supercell, whereas this number goes up to 83671050 within the 2x2x1 supercell. A three stage protocol was designed and used throughout this work in order to sample the configurational space of crystalline solids with random atomic arrangements. This scheme employed three levels of progressively more complex physical models to evaluate the energies of different configurations of NMTP system, which were used to fit Cluster Expansion (CE) model: Electrostatic Coulomb Energy (E_E) $\rightarrow$ Empirical pairwise interatomic potential (E_{FF}) $\rightarrow$ Density Functional Theory (PBE+U) (E_{DFT}) $\rightarrow$ Cluster Expansion approach energy (E_{CE}). The initial input structures for the exploration of compositional and configurational space were taken from the `materialsproject` database.[1] The reference codes for "pseudo

end members" are mp-6761, mp-1101704, and mp-1173671 for $NaTi_2(PO_4)_3$, $NaMnPO_4$, and Na_3PO_4, respectively. Supercell[2] package was used to generate all symmetrically non-equivalent configurations for different compositions using 2x1x1 supercell (rhombohedral representation). All generated configurations were ranked by their electrostatic Coulomb energy. Formal atomic charges *i.e.* Na^{+1}, Ti^{+4}, Mn^{+2}, P^{+5} and O^{-2}, scaled by a factor of 0.6 in order to mimic the screening effects, were used together with the Ewald summation method for estimating the electrostatic lattice energies. At each stoichiometry, 2000 structures corresponding to the lowest electrostatic energy were taken for further refinement using an empirical pairwise interatomic potential.[3] The functional form of the interatomic interaction potential comprised an electrostatic non-bonding term and a bonding term described by a Morse potential:

$$E_{ij} = D(1 - e^{-a_{ij}(r-r_0)})^2 + \frac{1}{4\pi\varepsilon_0}\frac{Z_i Z_j}{r} \tag{1}$$

The parameters for different species are reported in Table S1.[3]

Table S1: Interatomic pair potential (Equation 1) parameters for different species.

Atom	Z (scaled)	Bond	D	a	r_0	r_{min}	r_{max}
Na	+0.6	Na-O	0.023363	1.763867	3.006315	0.0000	3.000
Ti	+2.4	Ti-O	0.024235	2.254703	2.708943	0.0000	3.000
Mn	+1.2	Mn-O	0.029658	1.997543	2.852075	0.0000	3.000
P	+3.0	P-O	0.831326	2.585833	1.800790	0.0000	2.000
O	-1.2	O-O	0.042395	1.379316	3.618701	0.0000	3.500

All interatomic potential calculations were performed using the General Utility Lattice Program (GULP).[4] The cell parameters and atomic positions were relaxed before the final energy evaluation. Figure S1 shows the correlation plot between the electrostatic Coulomb and interatomic pair potential lattice energies for different configurations. One can see that simple electrostatic lattice energy is insufficient to find the lowest energy configurations as this additional refinement step by interatomic potential calculation is essential.

Density functional theory calculations

At each x, 20 2x1x1 (with an exception of 60 configurations at $x = 0.5$) and 10 2x2x1 configurations with the lowest energy found by the interatomic potential screening were selected for evaluation at the Density Functional Theory (DFT) level. Additional 10 2x1x1 randomly selected configurations were added at each stoichiometry to further extend the sampled space. The DFT calculations were carried out using the Vienna Ab Initio Simulation Package VASP.[5] Perdew-Burke-Ernzerhof (PBE) generalized gradient approximation (GGA) functional was used to describe the exchange-correlation effects and projector augmented wave formalism (PAW) was employed to treat the core electrons.[6–8] Standard sets were used for P and O atoms and p states were treated as valance for all metals. The electronic wavefunctions were expanded using plane waves with a kinetic energy cut-off of 520 eV. In order to properly describe the Mn d electrons and obtain the correct high-spin configuration, a rotationally invariant Dudarev LDA+U method as implemented in VASP was used.[9] The U_{eff} value of 3.9 eV was intentionally optimized in order to yield the high-spin Mn configuration which was monitored by evaluating the magnetic moment on Mn atoms. Monkhorst-Pack scheme together with the algorithm of Wisesa et al. were used to select the k-point mesh with a density of at least 30 $Å^{-1}$ for integrating the first Brillouin zone.[10,11] The lattice constants of all configurations were optimized by fitting the volume vs. energy data to the Birch-Murnaghan equation of state and adding an additional re-optimization step with a constant plane wave cut-off. The convergence criteria were set to 6x10^{-1} eV/f.u., 6x10^{-2} eV/f.u. and 6x10^{-3} eV/f.u. for the cell, atom positions and electronic degrees of freedom, respectively. Tetrahedron method was used for the smearing of occupied electronic states. Ionic relaxations were carried out using residual minimization method - direct inversion in the iterative subspace (RMM-DIIS) algorithm. The correlation plot between the interatomic pair potential and DFT PBE+U energies for sampled configurations is presented in Figure S2. It is apparent, that interatomic pair potential energies, at least in this system, are well correlated with DFT energies over a broad range of configurational space as indicated by random data points in Figure S2. However, the correlation is less pronounced for the lowest energy configurations, which limits the applicability of interatomic pair potentials

for fine energy sampling. Nevertheless, given that sufficiently large number of E_{FF} configurations are taken into consideration, one could expect to sample the most stable one. Indeed, even during Cluster Expansion driven Monte Carlo simulations, no configurations with lower potential energy (within 2x1x1 supercell) were found.

An additional refinement of structures laying on the convex hull was carried out by hybrid DFT using the CRYSTAL17 software suite.[12,13] This step was carried out within the linear combination of atomic orbitals (LCAO) formalism together with B1WC[14,15] hybrid exchange-correlation functional as implemented in the CRYSTAL17 suite.[12,13] All-electron Gaussian basis sets with triple-zeta valence with polarization functions on Na, Ti, Mn, P and O were used.[16] We used different supercells to calculate $Na_{1+2x}Mn_xTi_{2-x}(PO_4)_3$ at different x: 148 (for x = 0.25), 76 (for x = 0.5), 78 (for x = 0.75), 40 (for x = 1.0), 164 (for x = 1.25) atoms. The Monkhorst-Pack sheme was applied to map the k-points on a 8x8x8 (for x = 1.0) mesh whereas the k-points for other x were chosen accordingly.[10] The Coulomb and exchange integral tolerance factors 8, 8, 8, 8, 16 were used in the calculations together with the extra-large numerical integration grid. The energy convergence criterion was set to 10^{-8} a.u. for the self-consistent field (SCF) total energy and 10^{-7} a.u. for the optimization of lattice parameters and atomic positions. The same parameters were also used for the periodic hybrid DFT calculations of the Raman spectra. In order to better reflect the experimental conditions a temperature of 300 K and excitation laser frequency of 532 nm wre used in CRYSTAL17 calculations. The Lorentzian peaks were broadened by 3 cm^{-1} for $NaTi_2(PO_4)_3$ and 35 cm^{-1} for α-$Na_3MnTi(PO_4)_3$ in Figure 6. The influence of Lorentzian FWHM broadening parameter on the Raman spectrum of α-$Na_3MnTi(PO_4)_3$ is shown in Figure S3.

Cluster Expansion approach

Clusters Approach to Statistical Mechanics CASM code,[17] which automates the construction and parameterization of effective CE Hamiltonians and implements them in Monte Carlo simulations was used in this work.[18–20] The main idea of Cluster Expansion method is based on the assumption that it is possible to separate the original lattice atomic positions $(R_1,\ldots,R_M)$ into those which

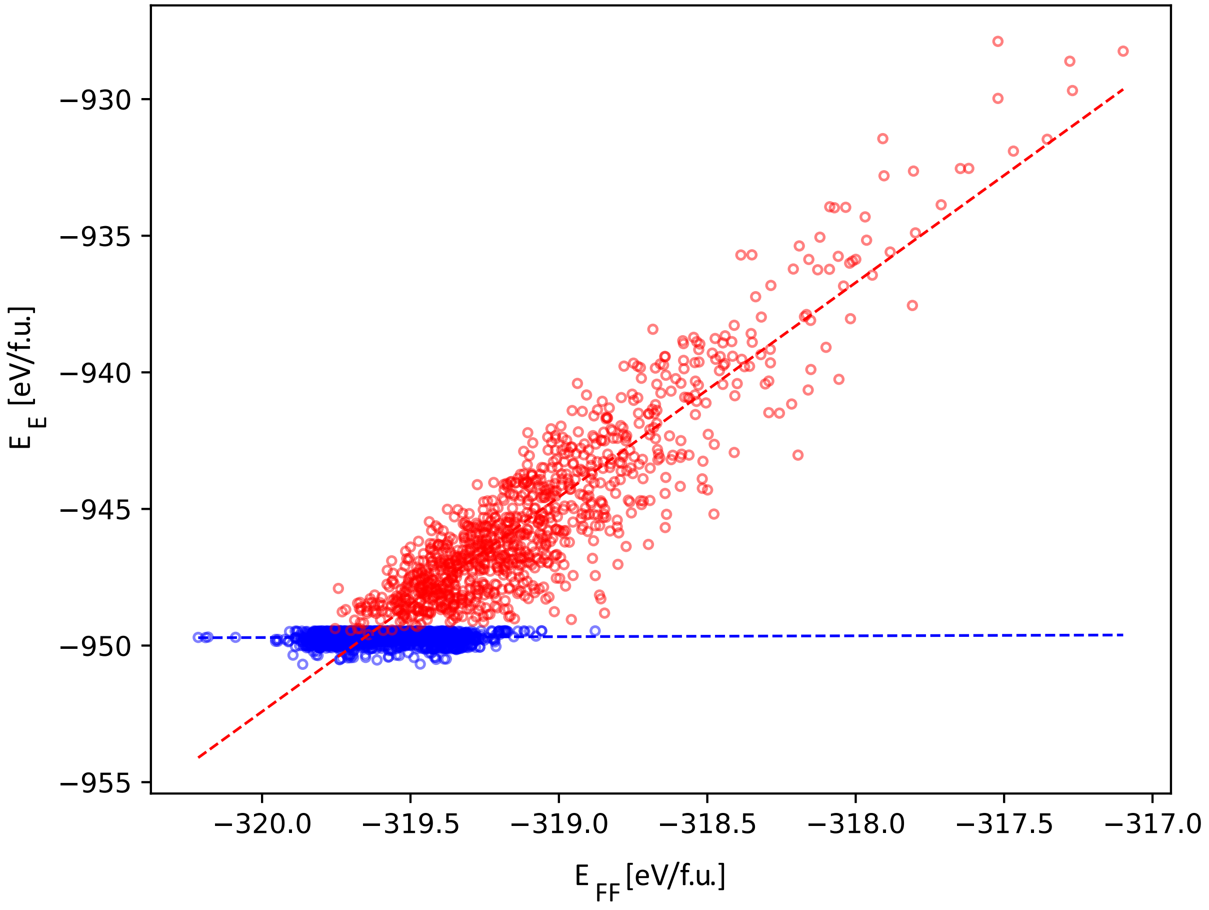

Figure S1: Correlation between electrostatic Coulomb and interatomic pair potential energies for different configurations in $Na_3MnTi(PO_4)_3$ (2x2x1 supercell). Blue and red circles represents 2000 lowest E_E and 1000 random configurations, respectively. Dotted lines marks corresponding linear regression fits.

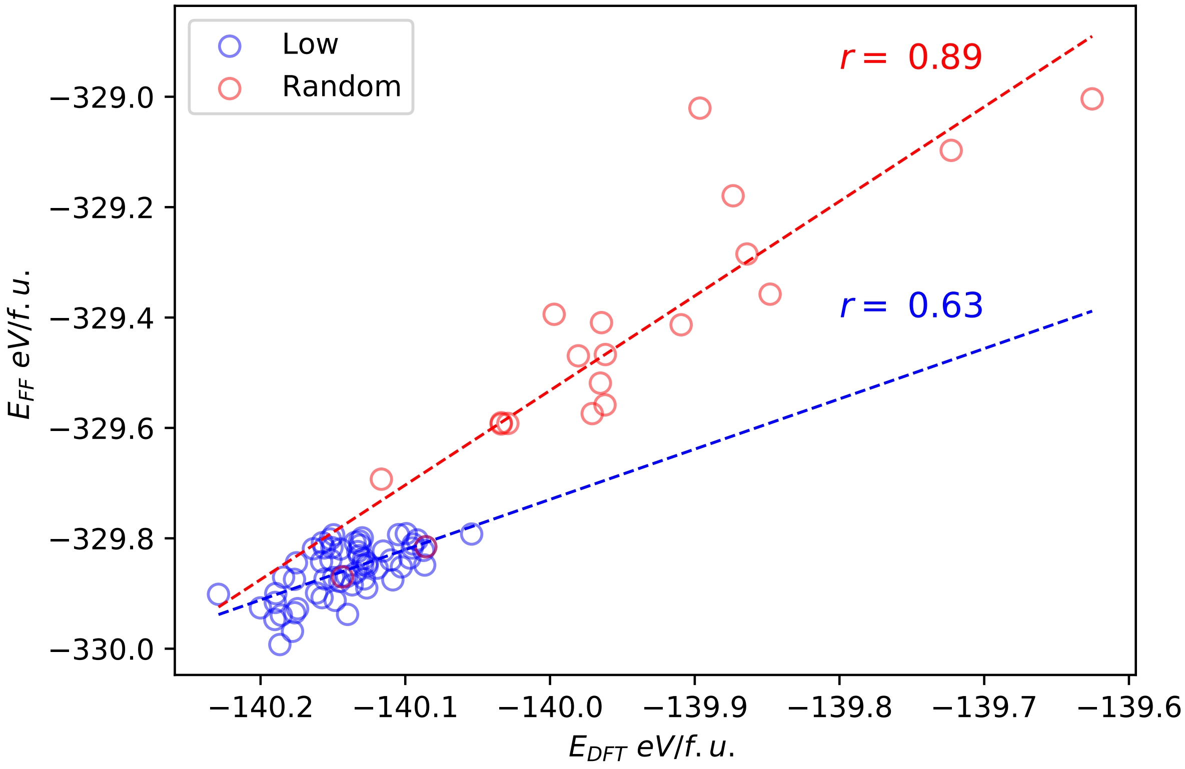

Figure S2: Correlation between interatomic pair potential energies and DFT PBE+U energies for different configurations in $Na_2Mn_{0.5}Ti_{1.5}(PO_4)_3$. Blue and red circles represents 60 lowest E_{FF} and 20 random configurations, respectively, Pearson's correlation coefficients and linear regression fits presented in corresponding colours.

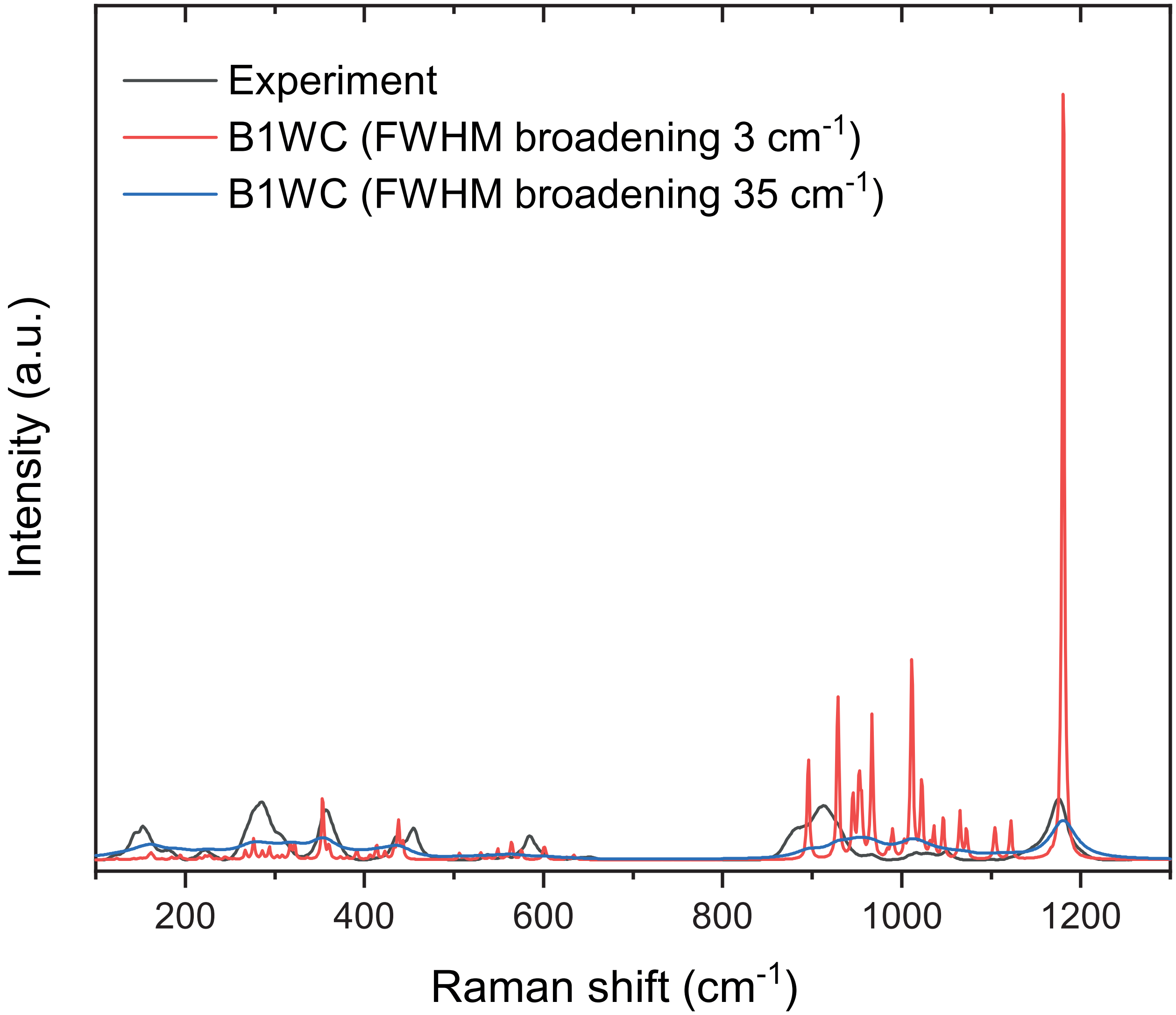

Figure S3: The influence of Lorentzian FWHM broadening parameter on the Raman spectrum of α-$Na_3MnTi(PO_4)_3$.

remain fixed and those where the atoms belonging to that position can be removed, added or replaced.[21] In this way one can focus on the changes in lattice energy (E) taking place upon these modifications which can be expressed as a simple function of the occupation of these sites by different species (Z). The energy function is then dimensionally reduced from a function of atom positions to that of a configuration vector which uniquely maps the occupation of specific lattice sites $(1,\ldots,M)$ by different species (Z):[22]

$$E(R_1,\ldots,R_M) \rightarrow E_{conf}(Z_1,\ldots,Z_M) \tag{2}$$

The occupation of each site is described by spin-like variables σ_i, which can for example be σ_i = +1 if site is occupied by Ti and σ_i = -1 if o occupied by Mn. In the same way, for Na site σ_i = +1 if it is occupied and σ_i = 0 if it is vacant. A specific configuration is then fully described by a M-dimensional vector of discrete σ_i values assigned to each lattice site:

$$\sigma \equiv (\sigma_1,\ldots,\sigma_M) \tag{3}$$

Therefore, the energy of each configuration can then be expressed as a function of this vector:

$$E(\sigma) \equiv E(\sigma_1,\ldots,\sigma_M) \tag{4}$$

In order to evaluate $E(\sigma)$ for all possible configurations σ in a computationally efficient way a basis set is needed. This basis set must effectively describe different types of interactions present in a system. In this context, this basis set is formed from *clusters*, comprised of particular combination of lattice sites $\alpha = (i,j,k,\ldots)$ such as pairs, triples, quadruplets, *etc.* describing two-body, three-body, four-body, *etc.* interactions, respectively. The complete expansion would include all possible types of α up to M-body interactions. However, this expansion converges relatively fast and usually few interaction terms *i.e.* clusters with more than triplet interactions and large radii have only small contribution to the total cluster expansion energy.

This way the energy for a lattice site can be expressed in terms of clusters as:

$$E(\sigma) = E_{CE}(\sigma) = \sum_{\alpha} J_{\alpha} \bar{\Pi}_{\alpha}(\sigma) \tag{5}$$

where the sum runs over all possible inequivalent clusters (α), J_{α} is the "effective interaction strength" associated with a cluster α, $\bar{\Pi}_{\alpha}$, is called the correlation matrix. This matrix contains spin-products which are related to the probability of finding the cluster α in configuration σ:

$$\bar{\Pi}_{\alpha}(\sigma) = \frac{1}{N_{\sigma}} \sum_{\beta \equiv \alpha} \prod_{i \in \beta} \sigma_i \tag{6}$$

where N_{σ} is a supercell size. The values of $\bar{\Pi}$ are an average over lattice sites and rotated/translated clusters on each of those sites ranging between -1 and +1. In this approach, there are exactly as many possible configurations σ as there are possible clusters α at any given lattice or the same number of basis functions as there are possible configurations.

The main task of CE approach is to find such α and J_{α} that the mean-square deviation between DFT calculated $E_{DFT}(\sigma)$ and $E_{CE}(\sigma)$:

$$MSD^2 = \frac{1}{N_{\sigma}} \sum_{\sigma} (E_{DFT}(\sigma) - E_{CE}(\sigma))^2 \tag{7}$$

is minimized. *MSD* typically arises due to the truncation of the number of clusters or the accuracy of the DFT computed energies used in the fit.

The quality of the model and its predictive power is measured by the cross-validation score:

$$CV^2 = \frac{1}{N_{\sigma}} \sum_{\sigma} (E_{\sigma} - E_{CE}^{(\sigma)})^2 \tag{8}$$

where $E_{CE}^{(\sigma)}$ where is the predicted energy of structure σ obtained from a least-squares fit of only $n-1$ structures that excludes structure σ from the fit, whereas the error is estimated for all n structures. Once an optimal and reliable set of clusters α with corresponding J_{α} is obtained, one can use an effective Monte Carlo search to find new configurations

Initially, 230 configurations with 2x1x1 supercell size, distributed over the entire Mn concen-

tration range ($x = 0.0 - 1.5$) optimized by DFT were selected for training the initial CE model. The cluster basis set was truncated at the quadruplet interactions with maximum interaction radii of 15.54 Å, 10.0 Å, and 6.0 Å for pairs, triplets and quadruplets, respectively. The complete set contained 679 clusters. However only those with the shortest interaction radii in periodically degenerate groups were selected, which reduced the number to 128.

Genetic algorithm was used for finding the clusters with the highest contributions to the total CE energy and optimizing their J_α. The parameters for this algorithm were as follows:

- The initial population contained 100 individuals. Each one of them was initiated by randomly selecting 35 clusters α with J_α obtained by the linear regression and the rest assumed $J_\alpha = 0$.
- Mutation probability was set to 0.1.
- Crossover probability was set to 0.5, with all individuals split into 3 groups from which only the 2 best are allowed to crossover in each iteration.
- The algorithm was started from different initial populations and repeated 100 times Each optimization was run for 150 iterations.
- Cross-validation score (CV) was used as a fitness metric for the individuals, and the data set in each iteration was split into 5 equal parts (randomly shuffled at each iteration) out of which 1 was used for validation.

In order to reduce exploitation of some clusters due to limited training set size, and to generate more stable configurations we used the fallowing workflow: The initial CE model, was then used to perform canonical metropolis Monte Carlo search at each x ($2000 \rightarrow 0$ K at every 5 K), until formation energy was converged within 0.001 $eV/f.u.$ (minimum and maximum number of passes were set to 10^4 and 10^5, respectively). At each x, configurations which were predicted to have $\Delta\Delta E_{CE} < 0$ $eV/f.u.$ (lower formation energy than the lowest previously enumerated one at specific x) were selected for further refinement at DFT PBE+U level of theory. Obtained results were

then added to the training data, followed by refitting of CE model. This procedure was iterated with increasing supercell size (up to 2x2x2) until no new candidate configurations (having formation energy $\Delta E_{CE} \leq -0.002$ $eV/f.u.$) were found. By this procedure, the total training set was expanded to 255 unique structures, and the new most stable (according to ΔE_{DFT}) configurations were found for x = 1.25 and 1.5.

37 clusters obtained during CE optimizations are presented in Figure S4. As one can see the obtained parameters are physically sensible because they converge in terms of distance and cluster size. The validity of our CE model is also illustrated in Figure S5 showing the correlation between DFT and CE calculated formation energies of different configurations in $Na_{1+2x}Mn_xTi_{2-x}(PO_4)_3$ (*n.b.* The outlier points were not excluded from the CE fit.).

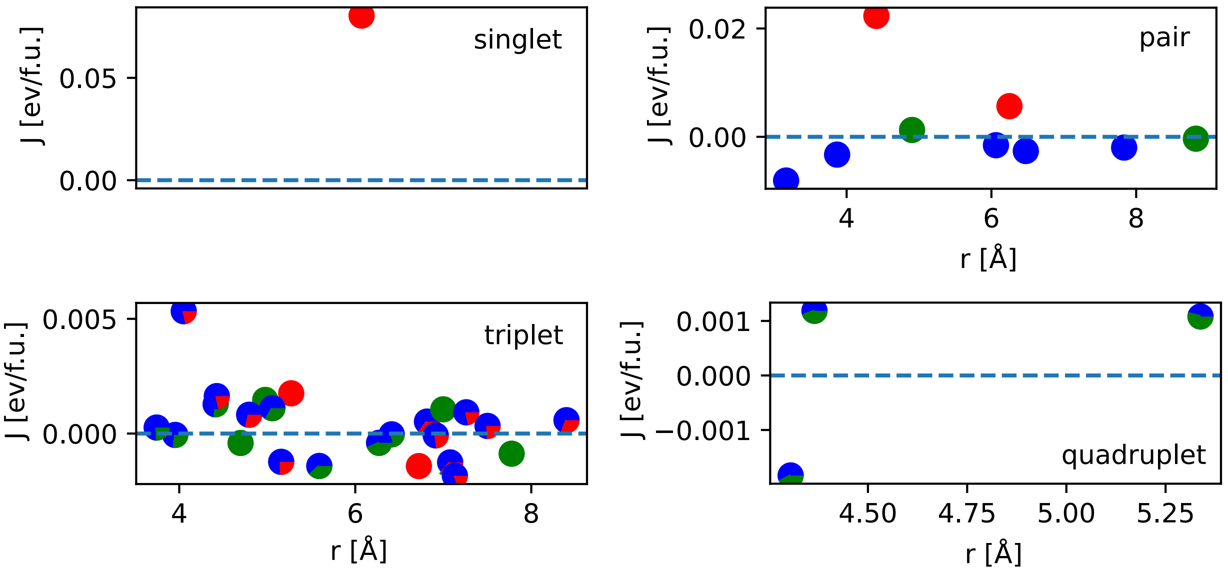

Figure S4: Effective interaction energy J_α (eV) dependence on average distance between lattice sites and multiplicity for selected clusters. For pairs and larger cluster the weights are estimated in terms of distances according to $w_j = \sum_{j=i} \frac{1}{d_j} / \sum_i \frac{1}{d_i}$. (green dots) Na/$V_{Na}$-Na/$V_{Na}$; (red dots) Ti/Mn-Ti/Mn; (blue dots) Na/V_{Na}-Ti/Mn. Singlets: (green dots) Na/V_{Na}; (red dots) Ti/Mn.

Calculation of phase diagram

The phase diagram was constructed by chemical potential (μ) driven semi-Grand Canonical Monte Carlo (SGCMC) simulations, where site occupations were varied for Mn and Na simultaneously

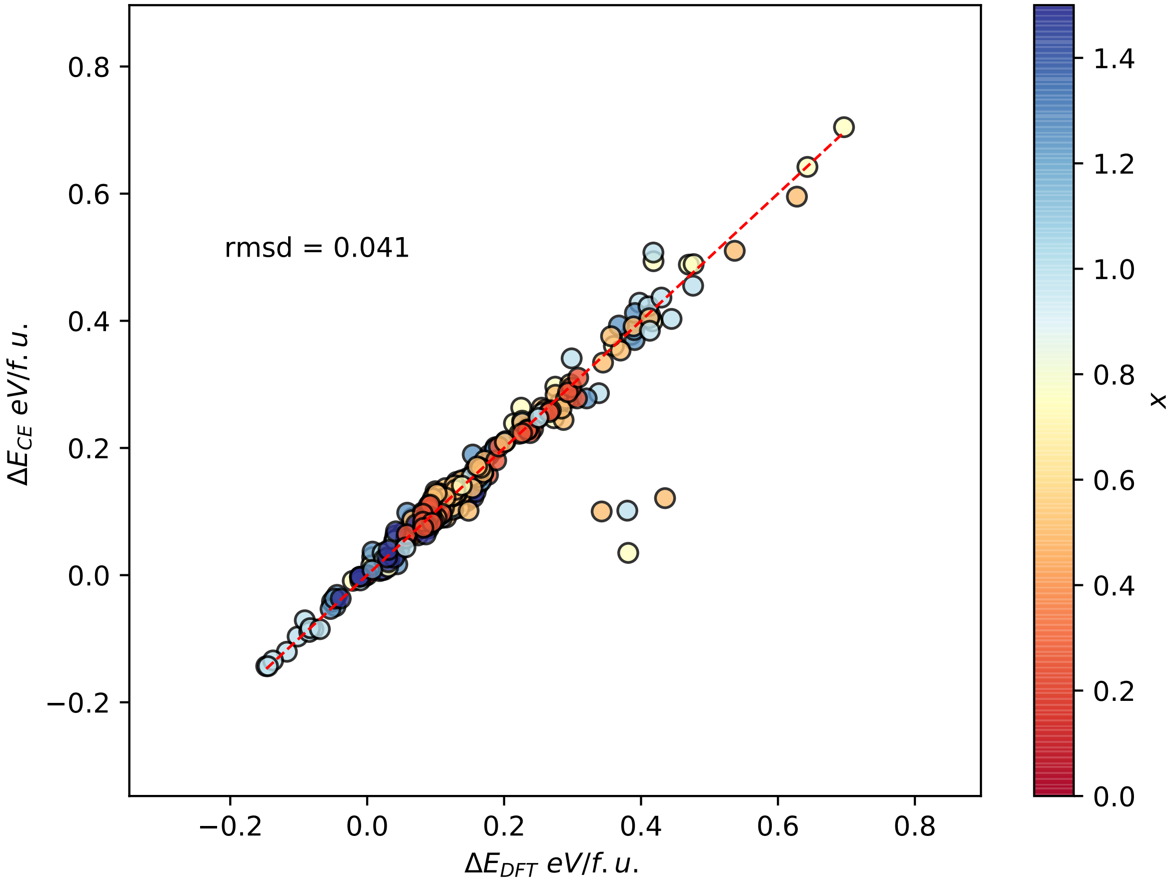

Figure S5: Correlation between DFT PBE+U and CE calculated formation energies for different configurations in $Na_{1+2x}Mn_xTi_{2\text{-}x}(PO_4)_3$. Color map corresponds to different *x* values. Dotted red line marks the ideal fit case.

in order to preserve charge neutrality. The SGCMC simulations were performed on a slightly in-house modified version of CASM, which was in turn based on the version recently made available by Deng *et. al.*[23] and available at (https://github.com/caneparesearch/CASMcode). The code was modified to make it possible to couple three sites which was necessary in this work. At each temperature from 5 K to 1200 K with ΔT = 10 K, the scans of chemical potential $-1 \leq \mu \leq 2$ with $\Delta\mu$ of 0.005 eV/f.u were performed in both directions starting from each configuration that lies directly on the convex hull, *i.e.* from x = 0 (-2.0 eV/f.u. $\rightarrow$ -1.0 eV/f.u.), from x = 1 (-1.0 eV/f.u. $\rightarrow$ -2.0 eV/f.u., and -1.0 eV/f.u. $\rightarrow$ 2.0 eV/f.u.), from x = 1.5 (2.0 eV/f.u.$\rightarrow$ -1.0 eV/f.u.). Phase diagram was constructed by numerically locating discontinuities in composition x with respect to chemical potential and mapping these points to composition-temperature domain, as illustrated in Figure S6. 10x10x10 sized supercell was used for SGCMC runs, 5x10^{-3} eV/f.u. formation energy and 10^{-2} x values were set as convergence criteria, and minimum and maximum passes were set to 500 and 5x10^{5}, respectively.

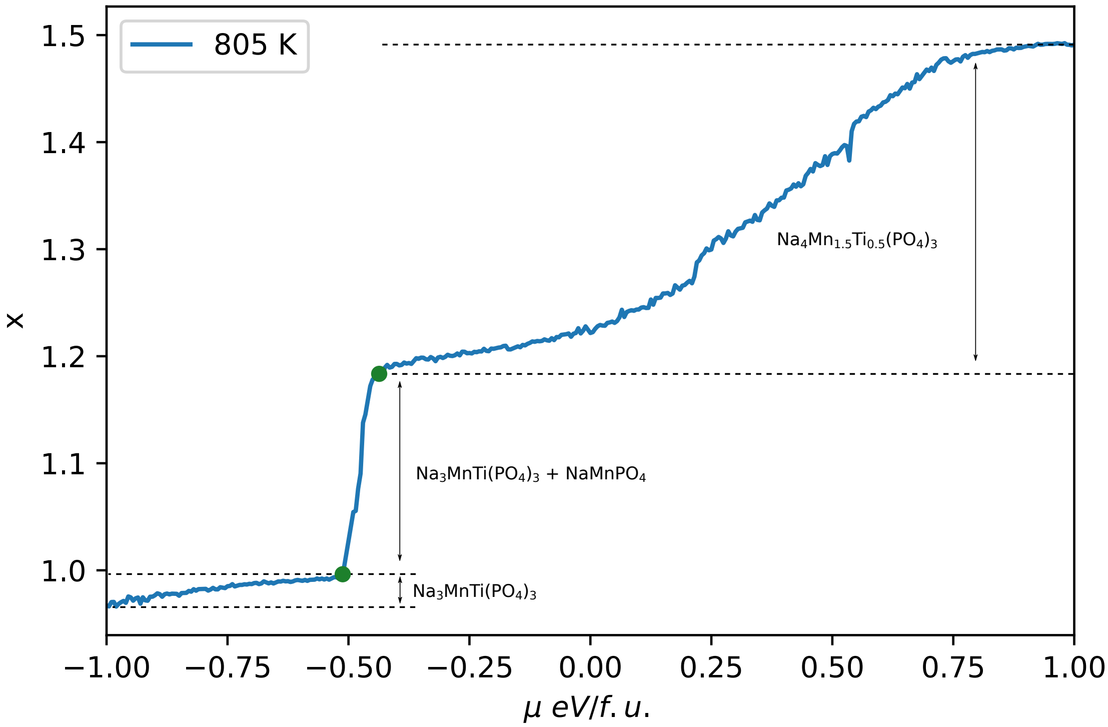

Figure S6: An example of SGCMC scan at 805 K with graphical representation of how the phase transitions are located and identified.

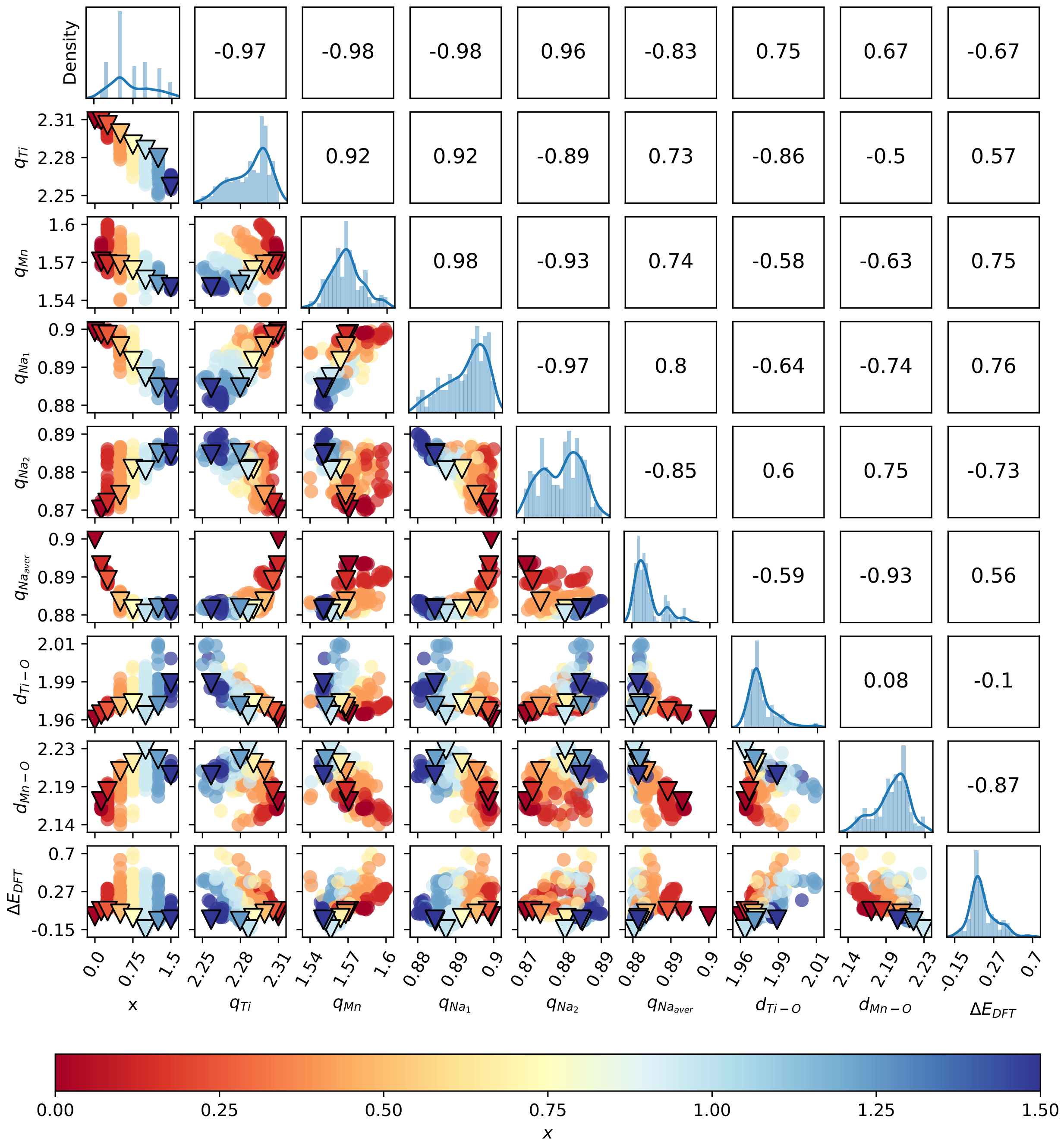

Figure S7: Correlation plots between different calculated atomic features in $Na_{1+2x}Mn_xTi_{2-x}(PO_4)_3$. Color map represents the x values, upper-right triangular section contains Pearson's correlation coefficients between the features of the lowest PBE+U energy structure at each x (marked by triangles). q_{Na_1}, q_{Na_2}, and $q_{Na_{aver}}$ are the atomic charges of sodium atoms at M2 and M1 positions in NASICON structure and their average, respectively. d_{Mn-O} and d_{Ti-O} are respective bond lengths. ΔE_{DFT} PBE+U formation energies. Atomic charges are obtained by Bader analysis, interatomic distances are evaluated from fully relaxed structures.

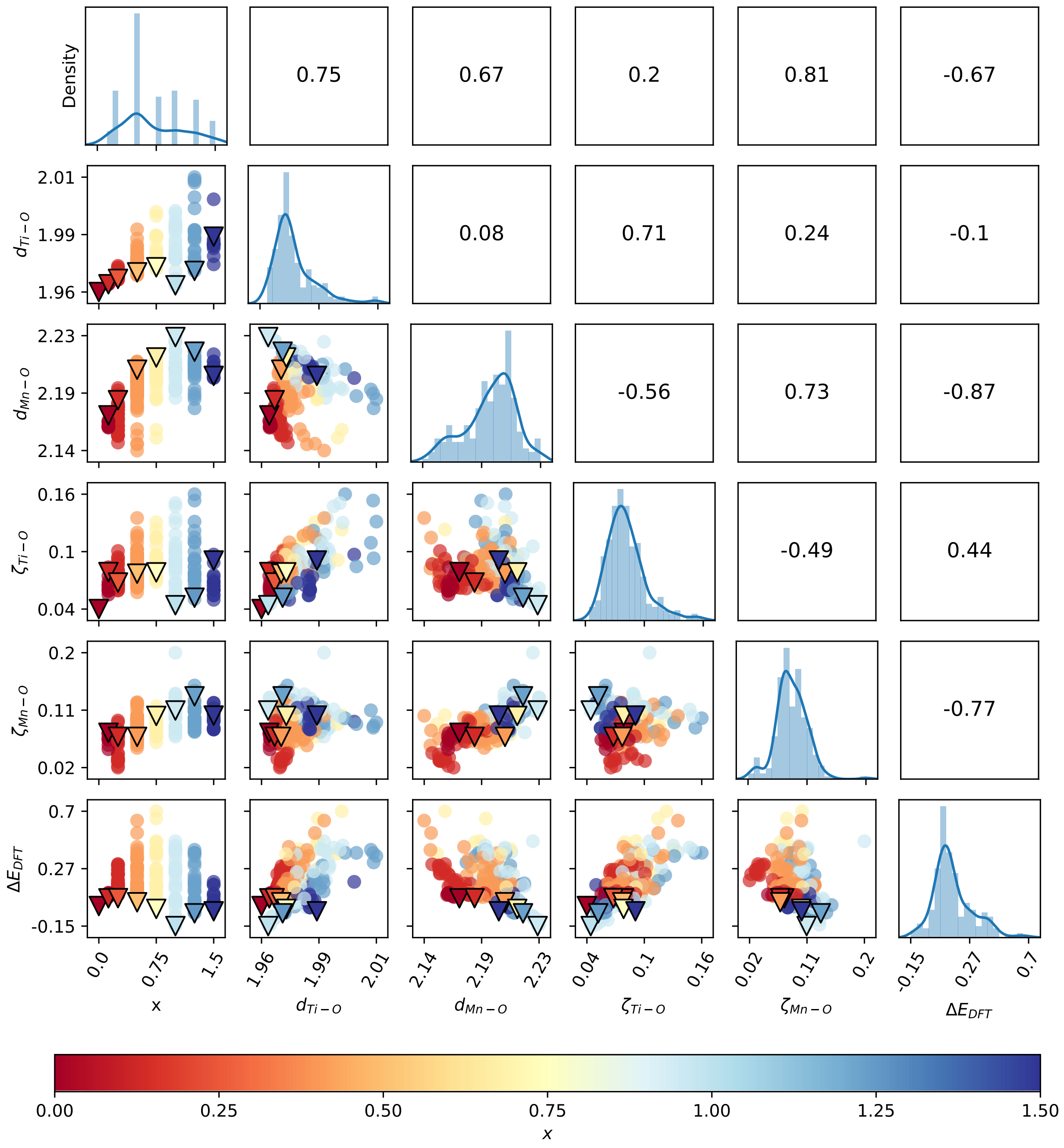

Figure S8: Correlation plots between different calculated atomic features in $Na_{1+2x}Mn_xTi_{2-x}(PO_4)_3$. Color map represents the x values, upper-right triangular section contains Pearson's correlation coefficients between the features of the lowest PBE+U energy structure at each x (marked by triangles). d_{Mn-O} and d_{Ti-O} are respective bond lengths. ζ is an octahedral distortion parameter defined as $\zeta_{Me-O} = \sum_{i=1}^{6} |d_i - d_{mean}|$, where d_i is a particular Me-O bond length in $[MeO_6]$ octahedron and d_{mean} is the arithmetic mean of all Me-O bond length in $[MeO_6]$ octahedron. ΔE_{DFT} PBE+U formation energies.

Experimental Details

X-Ray Diffraction

Table S2: Rietveld refined atomic positions for α-$Na_3MnTi(PO_4)_3$.

Atom	x	y	z	Occupancy	Wyckoff symbol	Uiso
Na1	0.000	0.000	0.000	1	2a	0.0152(31)
Na2	0.3180(9)	0.1627(26)	0.1422(12)	1	4c	0.0026(16)
Na3	0.000	0.2597(27)	0.5	1	2b	0.043(4)
Na4	0.2392(9)	0.8472(27)	0.2347(18)	1	4c	0.0283(11)
O1	0.147(2)	0.947(3)	0.637(4)	1	4c	0.031(4)
O2	0.4111(18)	0.957(3)	0.322(3)	1	4c	0.000
O3	0.5869(22)	0.782(5)	0.282(4)	1	4c	0.195(10)
O4	0.0445(22)	0.926(3)	0.791(4)	1	4c	0.069(6)
O5	0.7556(14)	0.942(3)	0.0193(28)	1	4c	0.000
O6	0.2569(18)	0.7691(30)	0.530(3)	1	4c	0.012(4)
O7	0.3528(15)	0.6807(30)	0.2082(26)	1	4c	0.017(3)
O8	0.1781(13)	0.0222(27)	0.3732(24)	1	4c	0.006(3)
O9	0.4248(13)	0.0664(23)	0.0318(21)	1	4c	0.000
O10	0.1636(28)	0.241(4)	0.141(5)	1	4c	0.080(7)
O11	0.0548(14)	0.6649(30)	0.4446(24)	1	4c	0.000
O12	0.0826(19)	0.7215(31)	0.161(3)	1	4c	0.019(4)
P1	0.1395(7)	0.0073(21)	0.7779(10)	1	4c	0.0113(16)
P2	0.000	0.5601(23)	0.500	1	2b	0.0077(27)
P3	0.000	0.6420(25)	0.000	1	2a	0.054(4)
P4	0.1553(7)	0.1870(24)	0.3099(12)	1	4c	0.0245(16)
Mn1	0.4030(7)	0.8492(22)	0.1009(14)	0.5	4c	0.0206(10)
Mn2	0.403	0.8492	0.1009	0.5	4c	0.0206
Mn3	0.1011(6)	0.8519(21)	0.3969(12)	0.5	4c	0.0287(11)
Mn4	0.1011	0.8519	0.3969	0.5	4c	0.0287
Ti1	0.4030(7)	0.8492(22)	0.1009(14)	0.5	4c	0.0206(10)
Ti2	0.403	0.8492	0.1009	0.5	4c	0.0206
Ti3	0.1011(6)	0.8519(21)	0.3969(12)	0.5	4c	0.0287(11)
Ti4	0.1011	0.8519	0.3969	0.5	4c	0.0287

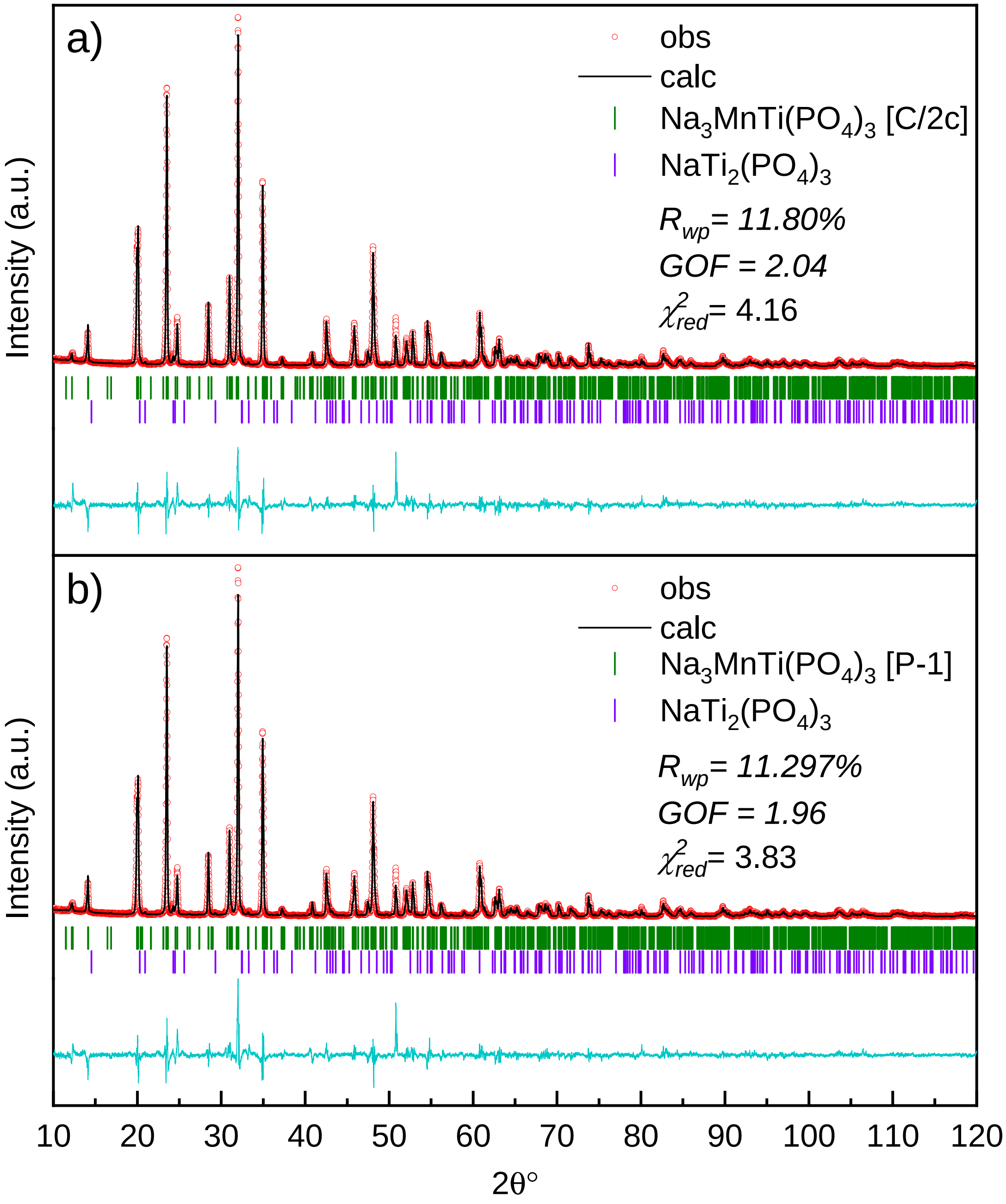

Figure S9: The results of Rietveld refinement of powder XRD pattern of $Na_3MnTi(PO_4)_3$ ($x = 1.0$) using (a) $C2/c$ and (b) $P\bar{1}$ space groups.

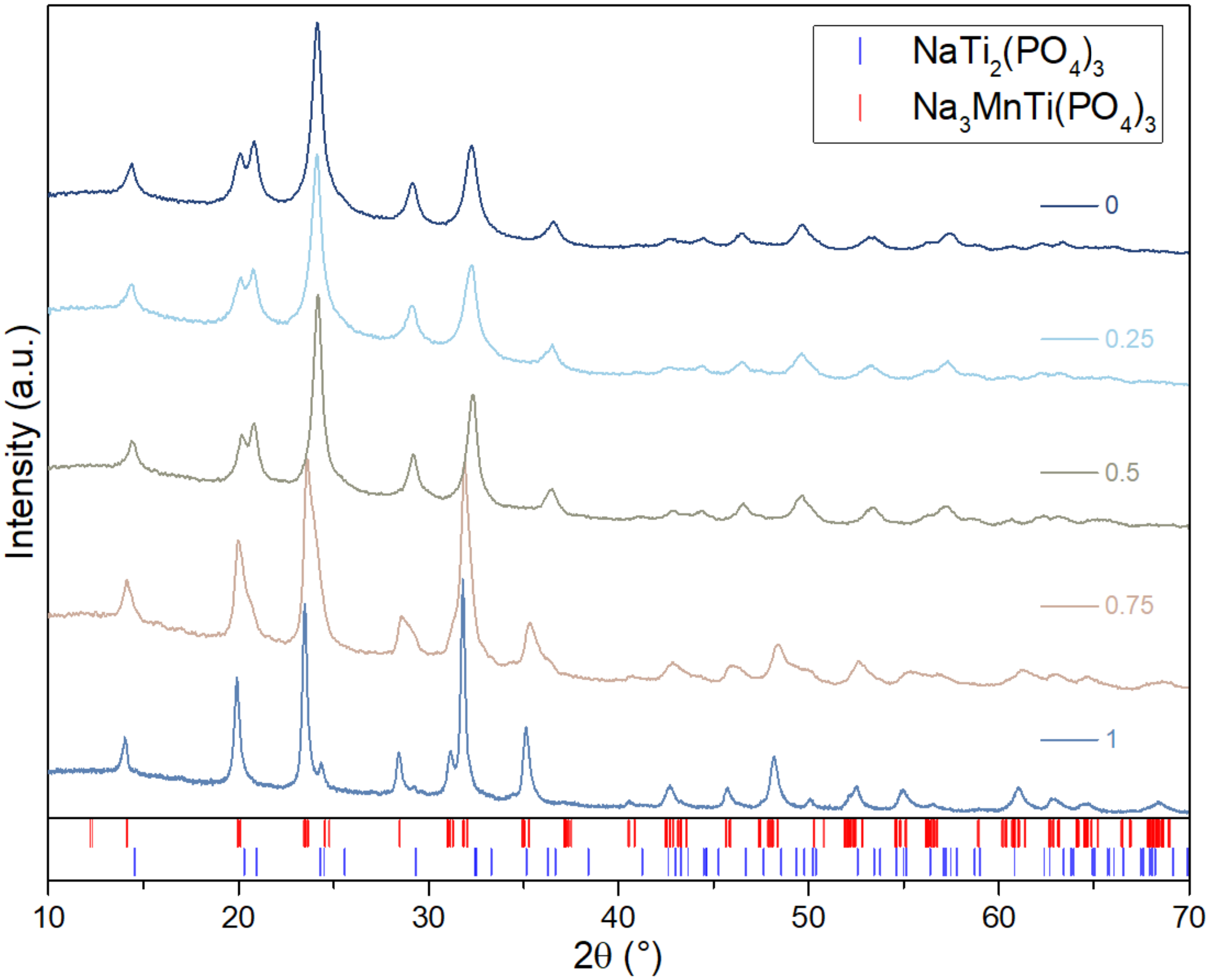

Figure S10: XRD patterns of $Na_{1+2x}Mn_xTi_{2-x}(PO_4)_3$ ($0 \leq x \leq 1$) prepared by sol-gel route

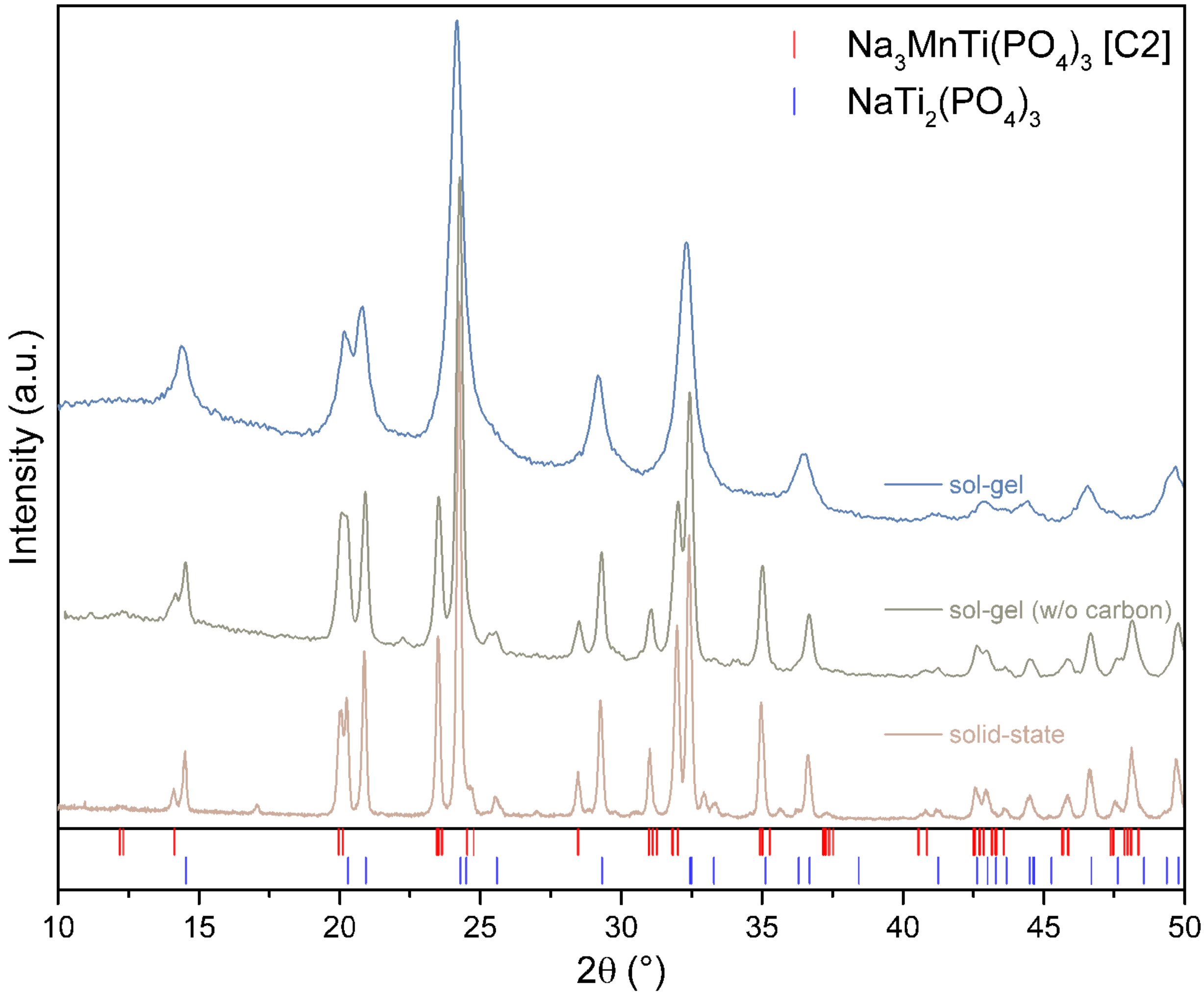

Figure S11: XRD patterns of $Na_{1+2x}Mn_xTi_{2-x}(PO_4)_3$ (x=0.5) prepared by solid-state and sol-gel routes

References

(1) Jain, A.; Ong, S. P.; Hautier, G.; Chen, W.; Richards, W. D.; Dacek, S.; Cholia, S.; Gunter, D.; Skinner, D.; Ceder, G.; Persson, K. A. The Materials Project: A materials genome approach to accelerating materials innovation. *APL Materials* **2013**, *1*, 011002.

(2) Okhotnikov, K.; Charpentier, T.; Cadars, S. Supercell program: a combinatorial structure-generation approach for the local-level modeling of atomic substitutions and partial occupancies in crystals. *Journal of cheminformatics* **2016**, *8*, 1–15.

(3) Pedone, A.; Malavasi, G.; Menziani, M. C.; Cormack, A. N.; Segre, U. A new self-consistent empirical interatomic potential model for oxides, silicates, and silica-based glasses. *The Journal of Physical Chemistry B* **2006**, *110*, 11780–11795.

(4) Gale, J. D.; Rohl, A. L. The general utility lattice program (GULP). *Molecular Simulation* **2003**, *29*, 291–341.

(5) Kresse, G.; Furthmüller, J. Efficient iterative schemes for ab initio total-energy calculations using a plane-wave basis set. *Physical review B* **1996**, *54*, 11169.

(6) Perdew, J. P.; Burke, K.; Ernzerhof, M. Generalized gradient approximation made simple. *Physical review letters* **1996**, *77*, 3865.

(7) Blöchl, P. E. Projector augmented-wave method. *Physical review B* **1994**, *50*, 17953.

(8) Kresse, G.; Joubert, D. From ultrasoft pseudopotentials to the projector augmented-wave method. *Physical review b* **1999**, *59*, 1758.

(9) Dudarev, S.; Botton, G.; Savrasov, S.; Humphreys, C.; Sutton, A. Electron-energy-loss spectra and the structural stability of nickel oxide: An LSDA+ U study. *Physical Review B* **1998**, *57*, 1505.

(10) Monkhorst, H. J.; Pack, J. D. Special points for Brillouin-zone integrations. *Phys. Rev. B* **1976**, *13*, 5188–5192.

(11) Wisesa, P.; McGill, K. A.; Mueller, T. Efficient generation of generalized Monkhorst-Pack grids through the use of informatics. *Physical Review B* **2016**, *93*, 155109.

(12) Dovesi, R.; Saunders, V.; Roetti, C.; Orlando, R.; Zicovich-Wilson, C.; Pascale, F.; Civalleri, B.; Doll, K.; Harrison, N.; Bush, I., et al. CRYSTAL17 User's Manual. University of Torino: Torino, 2017.

(13) Dovesi, R.; Erba, A.; Orlando, R.; Zicovich-Wilson, C. M.; Civalleri, B.; Maschio, L.; Rérat, M.; Casassa, S.; Baima, J.; Salustro, S., et al. Quantum-mechanical condensed matter simulations with CRYSTAL. *WIRE Comput. Mol. Sci.* **2018**, *8*, e1360.

(14) Wu, Z.; Cohen, R. E. More accurate generalized gradient approximation for solids. *Phys. Rev. B* **2006**, *73*, 235116.

(15) Bilc, D. I.; Orlando, R.; Shaltaf, R.; Rignanese, G. M.; Iniguez, J.; Ghosez, P. Hybrid exchange-correlation functional for accurate prediction of the electronic and structural properties of ferroelectric oxides. *Phys. Rev. B* **2008**, *77*, 165107.

(16) Peintinger, M. F.; Oliveira, D. V.; Bredow, T. Consistent gaussian basis sets of Triple-Zeta valence with polarization quality for solid-State Calculations. *J. Comput. Chem.* **2013**, *34*, 451–459.

(17) CASM, v0.3-dev. 2018; Available from https://github.com/prisms-center/CASMcode.

(18) Van der Ven, A.; Thomas, J.; Xu, Q.; Bhattacharya, J. Linking the electronic structure of solids to their thermodynamic and kinetic properties. *Mathematics and computers in simulation* **2010**, *80*, 1393–1410.

(19) Thomas, J. C.; Van der Ven, A. Finite-temperature properties of strongly anharmonic and mechanically unstable crystal phases from first principles. *Physical Review B* **2013**, *88*, 214111.

(20) Puchala, B.; Van der Ven, A. Thermodynamics of the Zr-O system from first-principles calculations. *Physical review B* **2013**, *88*, 094108.

(21) Hart, G. L. W.; Rosenbrock, C. W.; Bieniek, B.; Blum, V.; Richter, N. Hands-On Tutorial on Cluster Expansion. `http://helper.ipam.ucla.edu/publications/gss2014/gss2014_12176.pdf`.

(22) Sanchez, J. M.; Ducastelle, F.; Gratias, D. Generalized cluster description of multicomponent systems. *Physica A: Statistical Mechanics and its Applications* **1984**, *128*, 334–350.

(23) Deng, Z.; Sai Gautam, G.; Kolli, S. K.; Chotard, J.-N.; Cheetham, A. K.; Masquelier, C.; Canepa, P. Phase Behavior in Rhombohedral NaSiCON Electrolytes and Electrodes. *Chemistry of Materials* **2020**, *32*, 7908–7920.
